# Reprogramming Tumor‐Associated Macrophages via Targeted NAT10 Inhibition to Enhance Colorectal Cancer Immunotherapy

**DOI:** 10.1002/advs.202510854

**Published:** 2025-10-27

**Authors:** Jiahao Zhu, Benjie Xu, Rui Hu, Bo Yang, Peipei Shen, Yu Xu, Xiaojun Zhang, Danqi Qian, Gang Wu, Shengjun Ji, Yutian Zhao, Ke Gu

**Affiliations:** ^1^ Department of Radiotherapy and Oncology The Affiliated Hospital of Jiangnan University Wuxi Jiangsu 214000 P. R. China; ^2^ Department of Outpatient Chemotherapy Harbin Medical University Cancer Hospital Harbin Heilongjiang 150000 P. R. China; ^3^ Department of Radiotherapy and Oncology The Affiliated Suzhou Hospital of Nanjing Medical University Gusu School Nanjing Medical University Suzhou Jiangsu 215000 P. R. China

**Keywords:** colorectal cancer immunotherapy, macrophage polarization, NAT10 phase separation, sono‐sensitive biomimetic nanorobot targeting NAT10 phase‐separated condensates, SRSF2 N4‐acetylcytidine modification, SRSF2 Acetylation

## Abstract

This study introduces the Sono@NAT10 nanorobot for colorectal cancer (CRC) immunotherapy, designed to target NAT10 condensates in macrophages. Sono@NAT10, enclosed in a macrophage membrane using a metal–organic framework, shows stability under acidic conditions and releases NAT10 siRNA upon ultrasound activation. Flow cytometry and confocal imaging confirm effective macrophage targeting. Sequencing and proteomics reveal that NAT10 regulates SRSF2 protein stability, thereby promoting the transition of macrophages from the M2 to the M1 phenotype. In a CRC mouse model, Sono@NAT10 combined with anti‐PD‐1 (CD279) antibody inhibited tumor growth and enhances survival. These findings demonstrate the potential of Sono@NAT10 as a novel immunotherapeutic strategy for CRC by modulating NAT10 phase separation, providing a foundation for further exploration of its clinical application.

## Graphical abstract

This study presents the sono‐sensitive nanorobot Sono@NAT10, which targets NAT10 condensates to reprogram tumor‐associated macrophages in colorectal cancer. By reducing the acetylation level of SRSF2 protein and activating HDAC10, Sono@NAT10 promotes M1 polarization, suppresses tumor progression, and strengthens immunotherapy. The approach demonstrates safety, precise ultrasound‐triggered release, and significant synergy with immune checkpoint blockade, highlighting strong clinical translation potential.

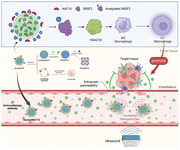

## 1 Introduction

Colorectal cancer (CRC) is one of the most common and deadly malignancies worldwide,^[^ ^1^ ^]^ exerting substantial effects on both patient survival and quality of life.^[^ ^2^ ^]^ Epidemiological data show that CRC represents the third most frequently diagnosed cancer and the fourth primary cause of cancer‐related mortality.^[^ ^3^, ^4^ ^]^ Conventional treatment strategies, including surgical resection, chemotherapy, and radiotherapy, provide limited survival benefits, particularly for patients with advanced CRC.^[^ ^3^, ^5^ ^]^ Recently, immunotherapy, especially the introduction of immune checkpoint inhibitors (ICIs), has emerged as a promising therapeutic approach for CRC.^[^ ^6^ ^]^ Despite encouraging clinical outcomes, immune evasion and low response rates continue to present major obstacles to effective treatment.^[^ ^7^ ^]^

In the tumor microenvironment (TME), macrophages serve as pivotal immune regulators with highly complex functions and diverse phenotypes.^[^ ^8^ ^]^ Based on their functions and cytokine secretion profiles, macrophages are generally classified into pro‐inflammatory M1 and anti‐inflammatory M2 subsets.^[^ ^9^ ^]^ In most malignant tumors, including CRC, M2 macrophages are predominant and contribute to tumor progression and metastasis by releasing pro‐tumor cytokines and growth factors.^[^ ^10^ ^]^ Therefore, reprogramming tumor‐associated macrophages from the M2 toward the M1 phenotype enhances tumor immune clearance,^[^ ^11^ ^]^ which has become a major focus in contemporary immunotherapy research.^[^ ^12^ ^]^

N‐acetyltransferase 10 (NAT10), a nucleoside acetyltransferase, has diverse biological functions, including regulation of the cell cycle, cell division, and ribosome biogenesis.^[^ ^13^ ^]^ Aberrant NAT10 expression has been observed in various cancers and is closely linked to tumor initiation and progression.^[^ ^14^, ^15^ ^]^ Notably, NAT10 participates in regulating multiple intracellular protein functions through forming phase‐separated condensates,^[^ ^16^ ^]^ providing a theoretical basis for its potential as a novel therapeutic target in cancer.^[^ ^17^ ^]^ In macrophage polarization, NAT10 may influence this process by modulating the modification and stability of key proteins.^[^ ^18^ ^]^

Biomimetic technology and sono‐sensitive nanorobots, as rapidly advancing drug delivery platforms, provide distinct advantages for targeted therapy and controlled release. By mimicking biological membrane structures, these nanorobots significantly enhance drug biocompatibility and targeting efficiency,^[^ ^19^ ^]^ while minimizing toxic side effects on normal tissues.^[^ ^20^ ^]^ In the field of cancer therapy, ultrasound (US)‐responsive release systems have opened new avenues for precision medicine.^[^ ^21^, ^22^ ^]^ These systems, guided and controlled by US waves, enable precise drug release at specific locations, maximizing therapeutic efficacy.^[^ ^22^, ^23^ ^]^

In this study, a sono‐sensitive biomimetic nanorobot targeting NAT10 phase‐separated condensates (Sono@NAT10) was developed. The nanorobot reduced the acetylation level of SRSF2 protein, thereby activating HDAC10 and reprogramming macrophages from the M2 phenotype to the M1 phenotype. Through this strategy, the immunotherapeutic efficacy of CRC was enhanced, providing more effective treatment options for patients. Enhancement of tumor immune responses through modulation of macrophage polarization represents a scientifically innovative approach with considerable clinical potential and may serve as a pivotal strategy for future cancer therapies.

## 2 Results

### 2.1 Successful Preparation of Sono@NAT10

Macrophage reprogramming is essential for modulating tumor immunity and improving immunotherapy efficacy.^[^ ^24^, ^25^ ^]^ To identify macrophage‐related drivers in CRC, we established a CRC mouse model (Figure S2A,B, Supporting Information). Tumor and adjacent normal tissues were collected from three mice, and macrophages were isolated using F4/80 and CD11b antibodies for RNA‐seq. Transcriptomic analysis revealed 272 upregulated and 103 downregulated genes(Figure S2C,D, Supporting Information). These 375 genes were analyzed using LASSO regression (Figure S2E, Supporting Information). Disease‐specific characteristic genes were screened using the support vector machine‐recursive feature elimination algorithm (Figure S2F, Supporting Information), while gene importance was evaluated with a random forest algorithm (Figure S2G, Supporting Information). This integrative approach identified one critical mRNA: NAT10 (Figure S2H, Supporting Information).

**Figure 1:**
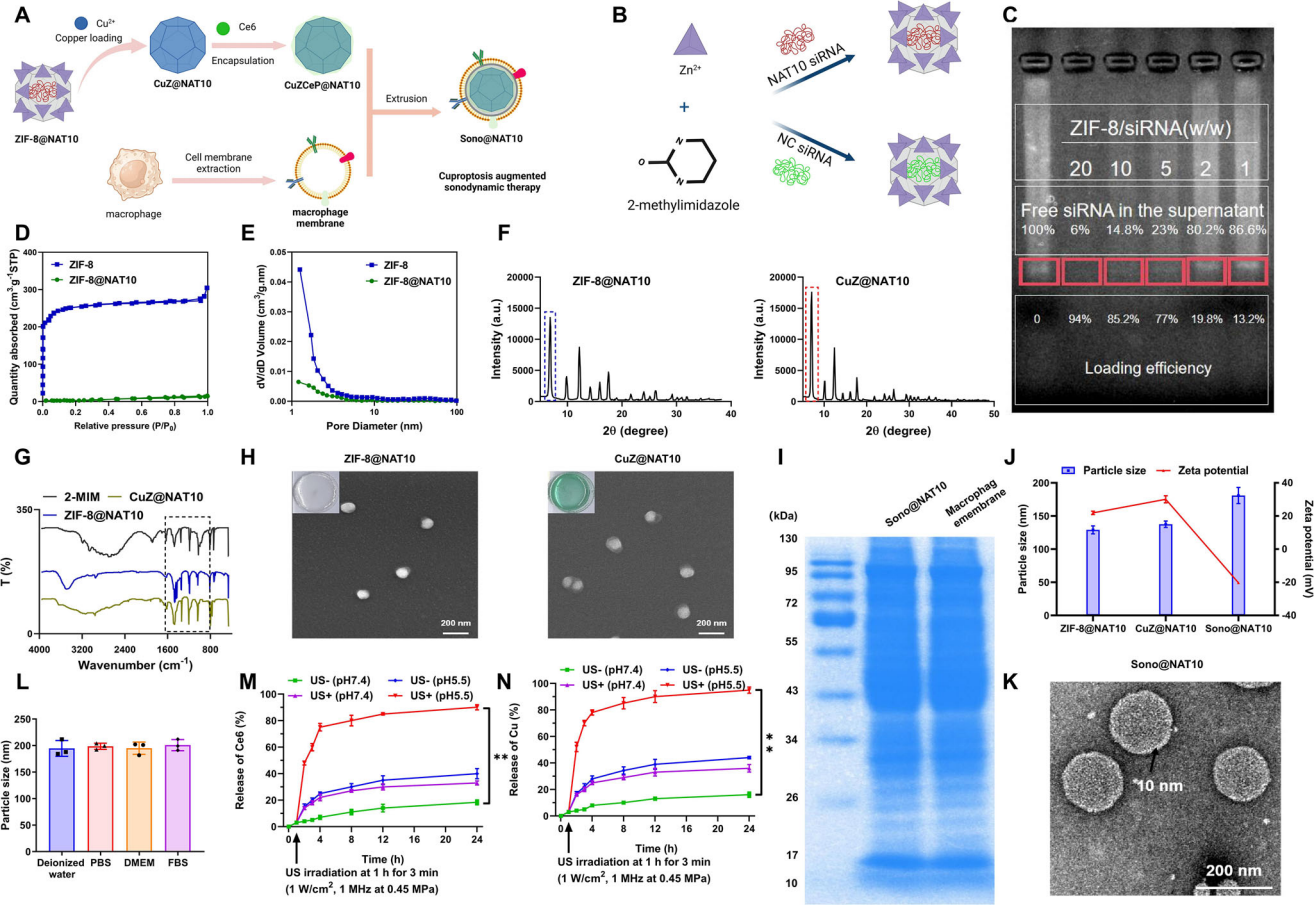
Synthesis and characterization of Sono@NAT10. Note: A) Schematic diagram of Sono@NAT10 synthesis (Created by Biorender); B) Schematic diagram of ZIF‐8@NAT10 and ZIF‐8@NC synthesis (Created by Biorender); C) Agarose gel electrophoresis showing the encapsulation efficiency of NAT10 siRNA in ZIF‐8@NAT10; D,E) Measurement of specific surface area (D) and pore size distribution (E) for ZIF‐8 and ZIF‐8@NC; F,G) X‐ray (F) and FT‐IR (G) characterization of ZIF‐8@NAT10 and CuZ@NAT10; H) SEM images of ZIF‐8@NAT10 and CuZ@NAT10; I) SDS‐PAGE analysis of macrophage membrane and Sono@NAT10 protein profiles; J) DLS analysis of zeta potential and particle size of Sono@NAT10; K) TEM images of Sono@NAT10; L) Particle size measurements of Sono@NAT10 in various media at 37 °C; M,N) Release profiles of Ce6 (M) and Cu (N) from nanoparticles under US stimulation and acidic/neutral pH conditions. Comparisons among different groups were performed using one‐way ANOVA, while comparisons of data from different groups at multiple time points were conducted using two‐way ANOVA. ** indicates *p <* 0.01 between groups. All experiments were repeated three times.

NAT10 has been demonstrated as a valuable prognostic indicator and an emerging therapeutic candidate in CRC.^[^ ^26^, ^27^ ^]^ Targeting NAT10 has enhanced cancer immunotherapy efficacy by mediating macrophage reprogramming.^[^ ^28^ ^]^ Therefore, developing nanomaterials targeting NAT10 in macrophages offers a novel treatment approach for CRC patients. In this study, we designed a nanomaterial capable of targeting macrophages within CRC tissues, delivering NAT10 siRNA, and amplifying immunotherapy outcomes (**Figure** **1**A). The nanomaterial encapsulates siRNA within a metal–organic framework (MOF) structure composed of Zn^2+^ and 2‐methylimidazole, effectively protecting the siRNA from degradation. The MOF framework is further modified with copper to enhance its catalytic activity, and a sono‐sensitive agent, Ce6, is coated on its surface to enable rapid siRNA release under US stimulation. Finally, macrophage membranes are isolated and used to encapsulate the modified MOF framework. This step provides the nanomaterial with a “camouflage” effect, leveraging the properties of macrophage membranes to evade immune recognition, thereby improving stability and targeting efficiency in vivo.

We successfully fabricated the NAT10 siRNA‐loaded MOF (Figure 1B). The siRNA encapsulation efficiency exceeded 90% and 85% at mass ratios of 1:20 and 1:10, respectively (Figure 1C). Compared to ZIF‐8, ZIF‐8@NAT10 exhibited reduced specific surface area and smaller pore size distribution (Figure 1D,E). Copper was subsequently loaded onto the surface of the MOF framework, and the structures of ZIF‐8@NAT10 and CuZ@NAT10 were characterized using XRD and FT‐IR (Figure 1F,G). Electron microscopy revealed that CuZ@NAT10 had a distinctive blue coloration, rough surface morphology, and an average diameter of ≈100 nm, whereas ZIF‐8@NAT10 displayed no visible coloration and a smooth surface (Figure 1H).

Next, Ce6, a sono‐sensitizer, and O_2_‐filled PFC were loaded into CuZ@NAT10 to enhance sonodynamic therapy synergistically. The nanoparticles were then coated with macrophage membranes to improve their targeting ability and stability. SDS‐PAGE analysis confirmed the protein profiles of the macrophage membrane and Sono@NAT10 (Figure 1I). Compared to ZIF‐8@NAT10 and CuZ@NAT10, Sono@NAT10 exhibited a significantly higher zeta potential and larger particle size (Figure 1J). TEM images showed well‐dispersed nanoparticles with an average diameter of ≈150 nm and a membrane thickness of ≈10 nm (Figure 1K). Sono@NAT10 maintained stability in various culture media for at least 48 h, indicating its suitability for in vitro and in vivo applications (Figure 1L). Under US stimulation (US^+^) in acidic environments, Sono@NAT10 rapidly released Ce6 and Cu (Figure 1M,N).

These findings confirm the successful synthesis of Sono@NAT10 and its robust physicochemical stability.

### 2.2 Sono@NAT10 Effectively Blocks NAT10 Phase‐Separated Condensate Formation

The biocompatibility of Sono@NAT10 was confirmed through a series of in vitro assays. CCK‐8 assays confirmed no cytotoxicity toward macrophages (Figure S3A, Supporting Information). Additionally, after co‐culturing macrophages with the nanomaterial for five days, Calcein‐AM and PI staining were used to distinguish live and dead cells (Figure S3B, Supporting Information). The staining results revealed that over 95% of the cells remained viable. Hemolysis tests further confirmed the excellent biocompatibility of Sono@NAT10, with hemolysis rates consistently below 3%, significantly lower than the widely accepted threshold of 5% (Figure S3C,D, Supporting Information). These findings indicate that the hemolytic effects of Sono@NAT10 are negligible, reinforcing its exceptional biocompatibility.

**Figure 2:**
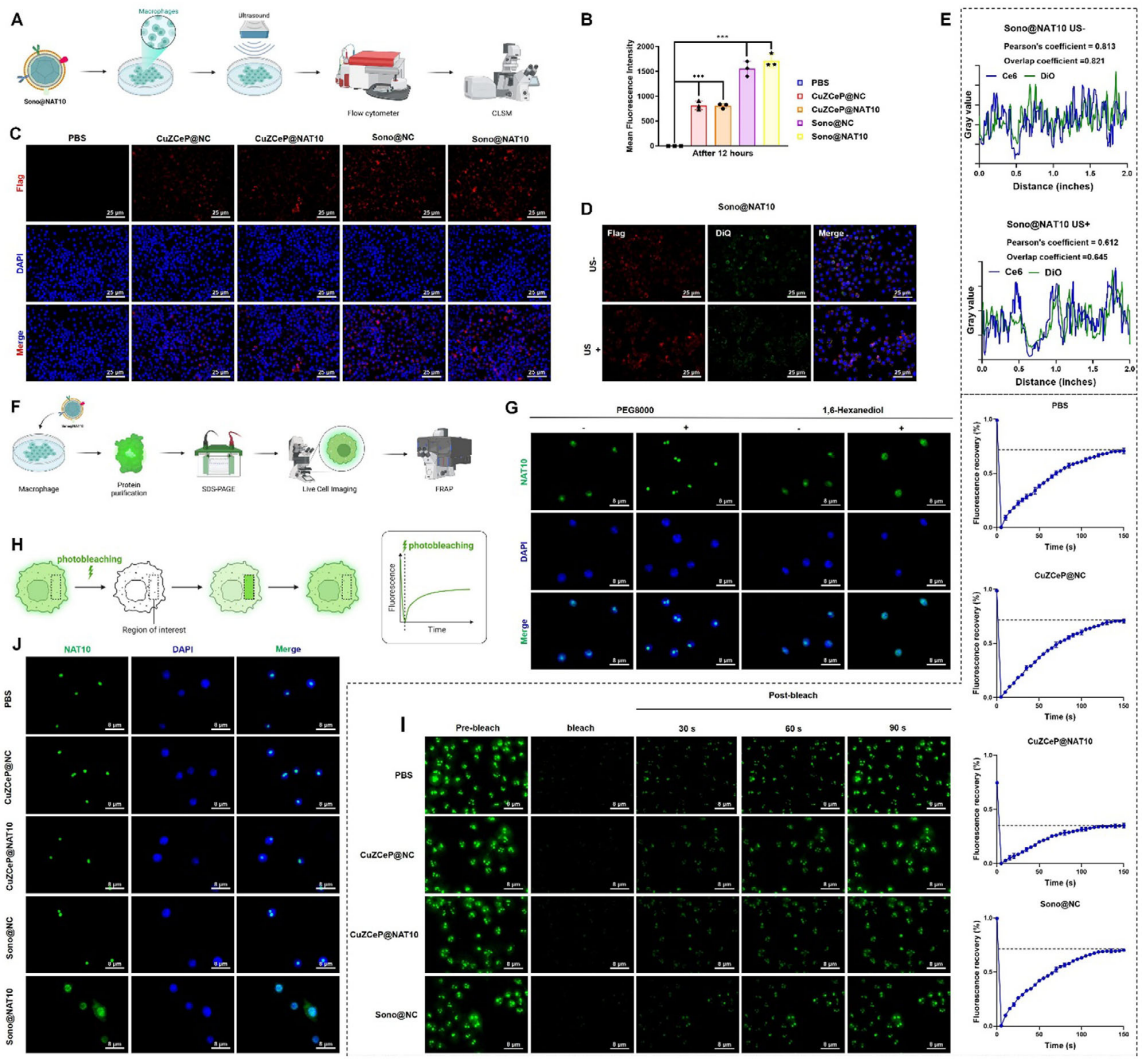
Impact of Sono@NAT10 on the NAT10 phase separation process. Note: A) Workflow of the cellular uptake experiment for Sono@NAT10 (Created by Biorender); B) Ce6 fluorescence intensity in macrophages detected via flow cytometry before US stimulation; C) Cellular uptake of nanoparticles by macrophages in different groups before US stimulation, scale bar = 25 µm; D,E) Fluorescence imaging (D) and grayscale analysis (E) of DiO‐labeled macrophage membranes before and after US stimulation, scale bar = 25 µm; F) Workflow for studying the NAT10 phase separation process in macrophages; G) Observation of NAT10 protein phase‐separated condensates after interference with PEG8000 or 1,6‐hexanediol, scale bar = 8 µm; H) Schematic of the FRAP experiment; I) Recovery of fluorescence condensates in macrophages treated with nanoparticles after photobleaching, scale bar = 8 µm; J) Formation of NAT10 protein condensates in different nanoparticle treatment groups, scale bar = 8 µm. Comparisons among different groups were performed using one‐way ANOVA. * indicates *p <* 0.05, *** indicates *p <* 0.001. All cellular experiments were repeated three times.

To evaluate cellular uptake, RAW264.7 macrophages were exposed to macrophage membrane‐coated nanocarriers (**Figure** **2**A). FCM data and CLSM images revealed rapid accumulation of Sono@NC and Sono@NAT10 in RAW264.7 cells even without US treatment, suggesting that macrophage membrane camouflage enhanced targeting efficiency (Figure 2B,C). To further assess the structural stability of Sono@NAT10 during cellular uptake, the macrophage membrane was labeled with DiO. As shown in Figure 2D,E, there was significant colocalization between Flag‐siRNA and DiO within cells, indicating that Sono@NAT10 maintained structural integrity during internalization. Upon mild ultrasound exposure, the colocalization decreased markedly, confirming ultrasound‐triggered structural disruption and efficient Ce6 release. Based on these results, subsequent functional assays were conducted under ultrasound stimulation.

NAT10 phase separation has been implicated in gastric cancer progression.^[^ ^29^ ^]^ To examine this process in vitro, we purified the GFP‐NAT10 protein and observed its phase separation using CLSM (Figure 2F; Figure S4A, Supporting Information). CLSM imaging revealed that PEG8000 treatment promoted condensate formation, whereas 1,6‐hexanediol, which disrupts hydrophobic interactions, abolished droplet assembly. These findings confirmed the presence of NAT10 phase separation in RAW264.7 cells (Figure 2G). To determine whether NAT10 undergoes phase separation in living cells, NAT10 was overexpressed in macrophages. Live‐cell imaging revealed nuclear condensate formation (Figure S4B, Supporting Information), which was enhanced by PEG8000 and disrupted by 1,6‐hexanediol (Figure S4C, Supporting Information), consistent with the in vitro assays. Furthermore, FRAP analysis demonstrated gradual fluorescence recovery of GFP‐NAT10 puncta within 90 s after photobleaching, a characteristic feature of phase‐separated condensates. These results collectively confirmed that NAT10 undergoes phase separation in vitro and live cells (Figure 2H,I).

We next examined how different nanomaterial formulations influence NAT10 phase separation in macrophages. In vitro, droplet formation assays revealed that the Sono@NAT10 group exhibited minimal fluorescence signals, whereas the CuZCeP@NC, CuZCeP@NAT10, and Sono@NC groups retained the ability to form droplets (Figure S4D, Supporting Information). Consistent results were observed in live cells, where condensates appeared in the nuclei of CuZCeP@NC, CuZCeP@NAT10, and Sono@NC groups, but were largely absent in the Sono@NAT10 group, which displayed dispersed nuclear and cytoplasmic localization (Figure 2J). Additionally, the FRAP experiment in Figure 2I demonstrated that after 90 s of photobleaching, the fluorescence recovery in the condensates formed by CuZCeP@NAT10 was ≈35%, while the fluorescence recovery in the condensates formed by CuZCeP@NC and Sono@NC was ≈70%. These results collectively suggest that Sono@NAT10 effectively inhibits the formation of NAT10 phase‐separated condensates.

### 2.3 Sono@NAT10 Reduces the Acetylation Level of SRSF2 Protein

**Figure 3:**
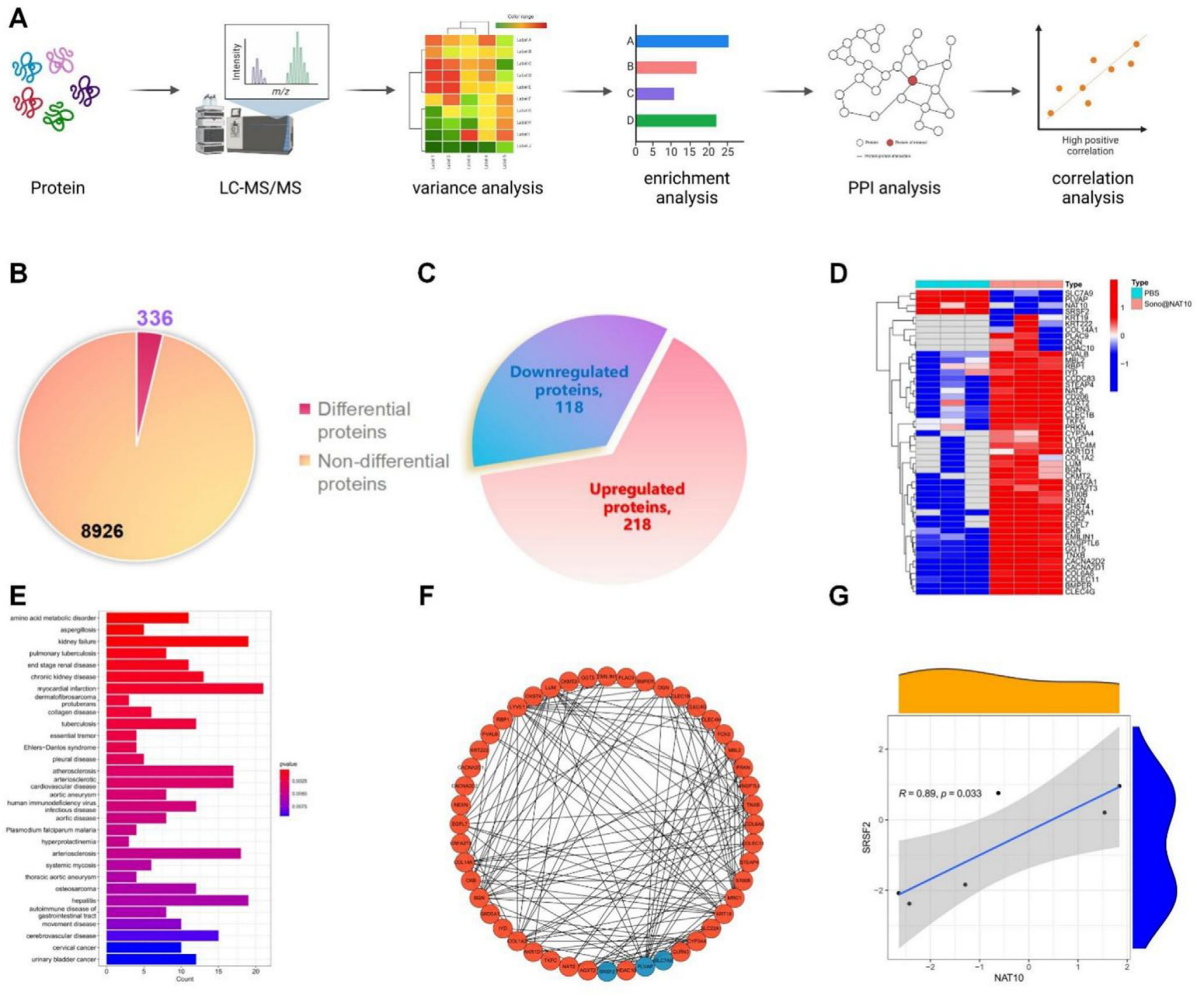
Proteomic sequencing analysis of macrophages after Sono@NAT10 treatment. Note: A) Workflow of the proteomic analysis (Created by Biorender); B) Proportion of DEPs identified between macrophages isolated from tumor tissues of 3 PBS‐treated mice and 3 Sono@NAT10‐treated mice; C) Comparison of upregulated (orange) and downregulated (blue) DEPs, *N* = 6; D) Heatmap displaying the differential expression of the top 30 DEPs in the proteomics dataset; E) DO enrichment analysis of DEPs; F) Interaction network of candidate DEPs, where each node represents a protein and connecting lines indicate PPI. Orange nodes represent upregulated proteins, and blue nodes represent downregulated proteins; G) Correlation analysis of NAT10 and SRSF2 protein levels in the proteomics dataset.

To explore downstream proteins regulated by NAT10, tumor tissues from PBS‐ and Sono@NAT10‐treated mice were collected. Macrophages were isolated using F4/80 and CD11b antibodies, and TMT‐based proteomic analysis was applied to assess differential protein abundance in the macrophage samples (**Figure** **3**A). A total of 9262 proteins were identified, with 336 DEPs detected in the Sono@NAT10 group (218 upregulated, 118 downregulated) (Figure 3B,C). Among the top 50 DEPs ranked by |log2FC| (Figure 3D), DO enrichment analysis highlighted 18 genes correlated with gastrointestinal diseases and cancers (Figure 3E). PPI analysis revealed that while most proteins were upregulated, SRSF2, PLVAP, and SLC7A9 were significantly downregulated(Figure 3F), with SRSF2 showing the strongest differential expression (Figure 3D). Furthermore, SRSF2 expression was significantly positively correlated with NAT10 protein levels (Figure 3G).

**Figure 4:**
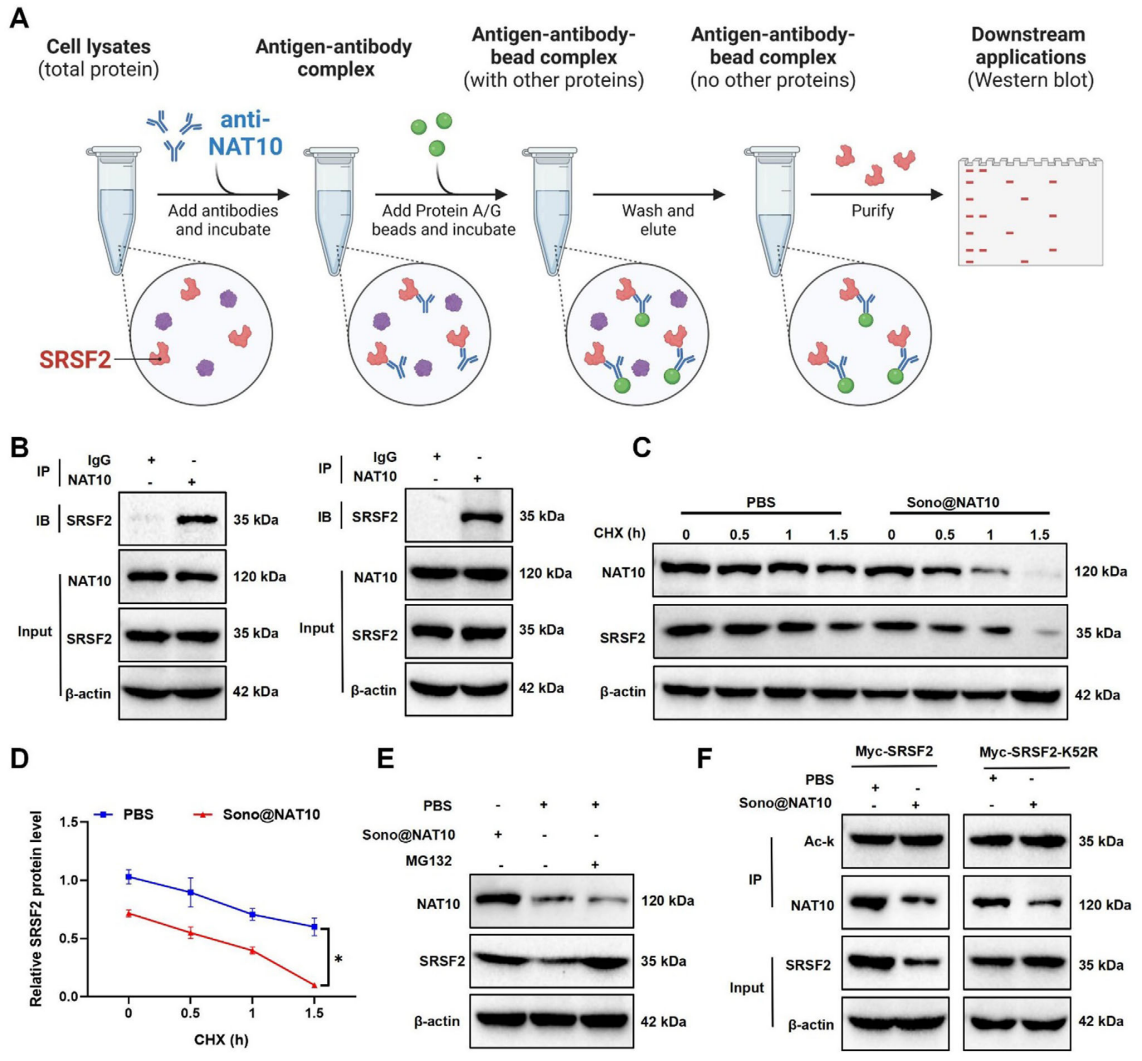
Interaction between NAT10 and SRSF2. Note: A,B) Workflow (A) (Created by Biorender) and results (B) of the Co‐IP experiment validating the interaction between NAT10 and SRSF2; C,D) Western blot analysis of NAT10 and SRSF2 protein levels in macrophages treated with the protein synthesis inhibitor CHX; E) Western blot analysis of SRSF2 protein expression after treatment with the proteasome inhibitor MG132; F) Acetylation analysis of SRSF2 regulated by NAT10, where Ac‐K (acetyl‐lysine) serves as a marker detected using specific antibodies. Comparisons of data from different groups at multiple time points were conducted using two‐way ANOVA. ** indicates *p <* 0.01. All cellular experiments were repeated three times.

Previous studies have shown that NAT10 enhances SRSF2 acetylation and stability via direct interaction.^[^ ^29^ ^]^ To validate this interaction, Co‐IP experiments confirmed the physical interaction between NAT10 and SRSF2 (**Figure** **4**A,B). IF analysis further revealed the nuclear colocalization of NAT10 and SRSF2 (Figure S5A,B, Supporting Information). Notably, Sono@NAT10 did not affect SRSF2 mRNA levels (Figure S5B, Supporting Information). However, CHX chase assays showed accelerated degradation of SRSF2 protein in the Sono@NAT10 group compared with PBS controls. This finding indicates that Sono@NAT10 reduces SRSF2 stability and promotes degradation (Figure 4C,D).

Proteasome inhibition with MG132 restored SRSF2 levels, supporting a NAT10‐dependent regulatory mechanism (Figure 4E). To elucidate the regulatory mechanism, wild‐type SRSF2 and an acetylation‐deficient mutant (SRSF2‐K52R) were expressed and validated (Figure S5C,D, Supporting Information). In vitro acetylation assays revealed that NAT10 knockdown significantly reduced SRSF2 acetylation levels but did not affect SRSF2‐K52R expression (Figure 4F), confirming that NAT10 mediates SRSF2 acetylation. These results demonstrate that Sono@NAT10 reduces the acetylation level of SRSF2 protein by modulating NAT10 phase‐separation condensate formation.

### 2.4 Sono@NAT10 Activates HDAC10 Protein Expression in Macrophages

**Figure 5:**
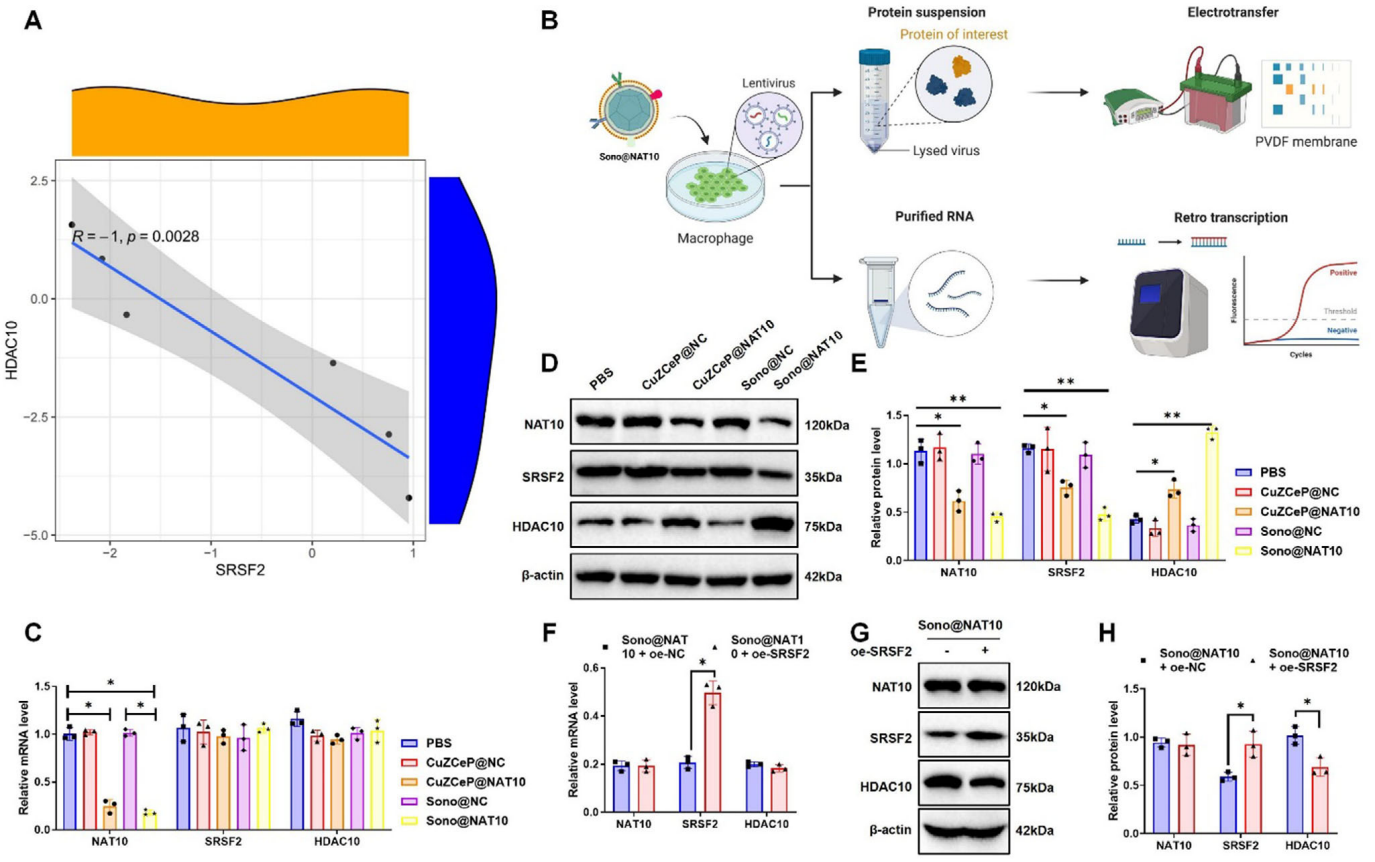
Impact of Sono@NAT10 on HDAC10 protein expression in macrophages. Note: A) Correlation analysis between SRSF2 and HDAC10 protein levels based on proteomic data; B) Experimental workflow investigating the relationship between SRSF2 and HDAC10 (Created by Biorender); C) RT‐qPCR analysis of NAT10, SRSF2, and HDAC10 mRNA expression levels after nanomaterial treatment; D,E) Western blot analysis of NAT10, SRSF2, and HDAC10 protein expression levels following nanomaterial treatment; F) RT‐qPCR analysis of NAT10, SRSF2, and HDAC10 mRNA expression levels after nanomaterial and lentiviral transfection treatments; G,H) Western blot analysis of NAT10, SRSF2, and HDAC10 protein expression levels following nanomaterial and lentiviral transfection treatments. All cellular experiments were repeated at least three times, with values presented as mean ± standard deviation. Comparisons between two groups were performed using independent samples *t*‐tests, while comparisons among different groups were conducted using one‐way ANOVA. * indicates *p <* 0.05; ** indicates *p <* 0.01.

SRSF2 is a splicing factor that recognizes m5C‐modified mRNAs, thereby regulating alternative splicing and protein expression^[^ ^30^ ^]^ Figure 3F indicates a co‐expression relationship between SRSF2 and the highly differentially expressed HDAC10 protein, with proteomics sequencing revealing a significant negative correlation between the two (**Figure** **5**A; Figure S6A,B, Supporting Information). Based on these findings, we hypothesized that HDAC10 might be a downstream regulatory target of SRSF2. To validate this hypothesis, we conducted experiments confirming the upstream–downstream relationship between SRSF2 and HDAC10 (Figure 5B). Initially, we verified the efficiency of lentivirus‐mediated SRSF2 overexpression, and the results demonstrated successful transfection with robust expression of SRSF2 (Figure S6C–E, Supporting Information).

Treatment with nanomaterials significantly altered NAT10 expression at both the mRNA and protein levels in macrophages from the CuZCeP@NAT10 and Sono@NAT10 groups compared with PBS, CuZCeP@NC, and Sono@NC groups. In contrast, SRSF2 and HDAC10 exhibited changes only at the protein level, without significant mRNA alterations (Figure 5C–E). Furthermore, overexpression of SRSF2 in macrophages markedly attenuated the effect of Sono@NAT10 on HDAC10 protein expression, confirming HDAC10 as a downstream effector of SRSF2 (Figure 5F–H). These findings demonstrate that Sono@NAT10 indirectly regulates HDAC10 expression in macrophages by modulating the acetylation level of SRSF2.

### 2.5 Sono@NAT10 Promotes Reprogramming of M2 Macrophages to the M1 Phenotype

**Figure 6:**
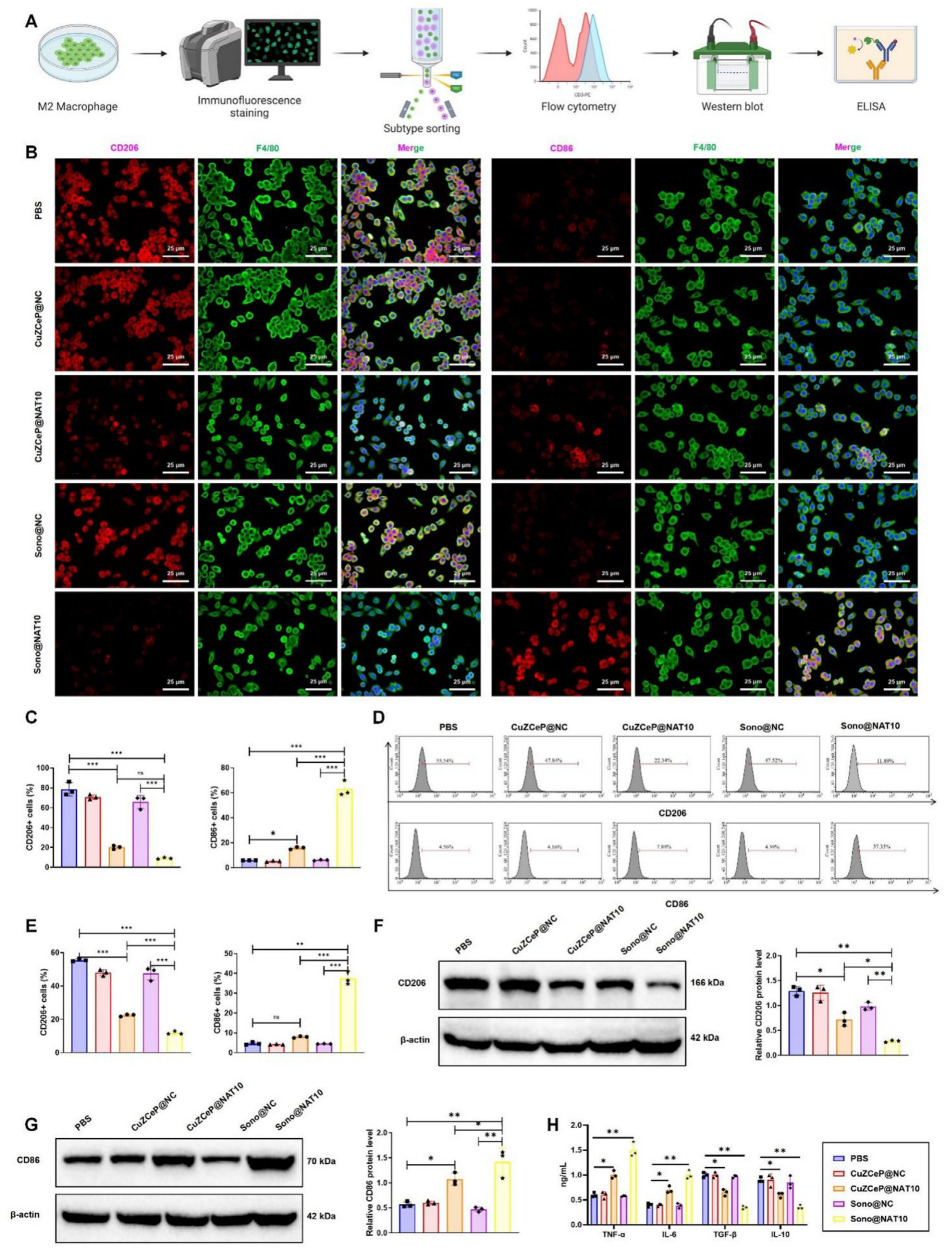
Regulatory effects of Sono@NAT10 on macrophage polarization. Note: A) The schematic diagram of the M2 macrophage reprogramming experimental procedure (Created by Biorender); B) IF staining showing the levels of M1 macrophage markers (CD86) and M2 macrophage markers (CD206) across groups, scale bar = 25 µm; C) Quantitative analysis of IF staining results; D) Flow cytometry analysis of CD86‐positive and CD206‐positive macrophages in each group; E) Quantitative analysis of flow cytometry results; F,G) Western blot analysis of CD86 (M1 macrophage marker) and CD206 (M2 macrophage marker) protein expression levels; H) ELISA analysis of inflammatory cytokine levels in the supernatant of co‐cultured cells across groups. Comparisons among different groups were conducted using one‐way ANOVA * indicates *p <* 0.05; ** indicates *p <* 0.01. All cellular experiments were repeated three times.

HDAC10 has been reported to facilitate the reprogramming of M2 macrophages to the M1 phenotype, thereby suppressing cancer progression.^[^ ^31^, ^32^ ^]^ To assess the effect of different nanoparticle treatments, macrophage polarization was examined by FCM and IF staining (**Figure** **6**A). Compared with PBS, CuZCeP@NC, and Sono@NC groups, treatments with CuZCeP@NAT10 and Sono@NAT10 markedly reduced M2 polarization while enhancing M1 markers, indicating a stronger reprogramming capability (Figure 6B–E). These findings suggest that Sono@NAT10 indirectly regulates HDAC10 expression, thereby influencing the polarization and reprogramming of M2 macrophages. Western blot analysis further corroborated these results (Figure 6F,G).

Compared to the PBS, CuZCeP@NC, culture supernatants from Sono@NAT10‐treated macrophages exhibited elevated pro‐inflammatory cytokines (TNF‐α, IL‐6) and reduced anti‐inflammatory cytokines (TGF‐β, IL‐10), confirming its potent immunostimulatory activity(Figure 6H). These findings demonstrate that Sono@NAT10 exerts no notable cytotoxic effects on macrophages while effectively promoting the reprogramming of M2 macrophages to the M1 phenotype.

### 2.6 Sono@NAT10 Suppresses CRC Cell Malignant Behavior

**Figure 7:**
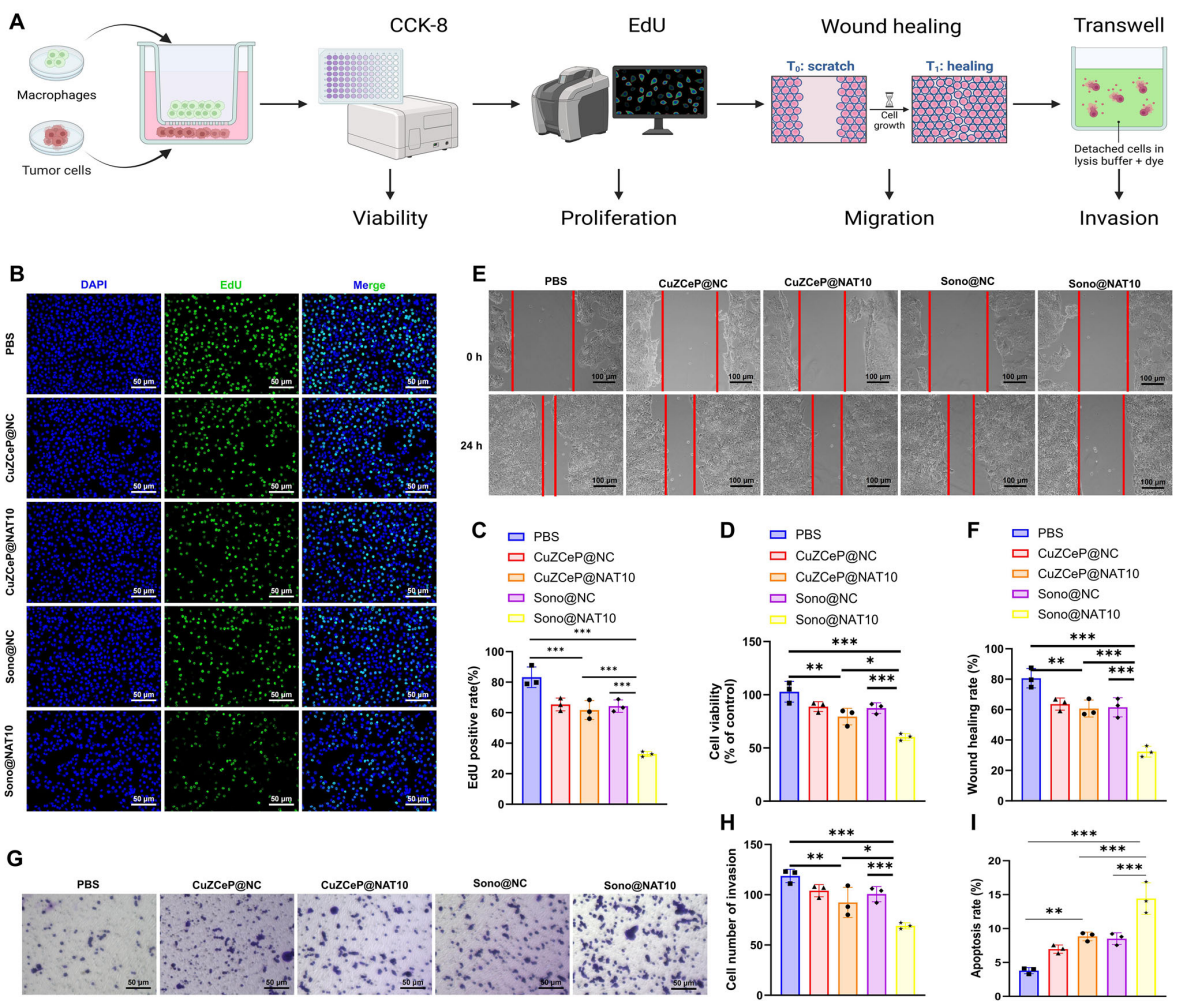
Effects of Sono@NAT10 on the behavior of CT26 cells. Note: A) Experimental workflow of Figure 7 (Created by Biorender); B,C) EdU assay to evaluate the proliferation capacity of CT26 cells across groups (scale bar: 50 µm); D) CCK‐8 assay to measure cell viability in CT26 cells; E,F) Scratch assay to assess the migration ability of CT26 cells across groups (scale bar: 100 µm); G,H) Transwell assay to analyze the invasion capability of CT26 cells (scale bar: 50 µm); I) Flow cytometry to detect apoptosis rates in CT26 cells across groups. Comparisons among the groups were performed using one‐way ANOVA, and post hoc tests within groups were conducted using the Tukey method. * indicates *p <* 0.05; ** indicates *p <* 0.01. All experiments were repeated three times.

To evaluate the role of macrophage reprogramming in CRC progression, macrophages pretreated with different nanomaterials and CRC cells (CT26 or CMT93) were co‐cultured (**Figure** **7**A). Functional assays were conducted to assess tumor cell behavior, including viability (MTT), proliferation (EdU), migration and invasion (wound healing and Transwell), and apoptosis (FCM). Compared to the PBS, CuZCeP@NC, and Sono@NC groups, nanomaterial‐treated groups markedly suppressed CRC cell viability, proliferation, migration, and invasion, while enhancing apoptosis. Among the treatments, Sono@NAT10 demonstrated the most pronounced effects, significantly improving therapeutic efficacy (Figure 7B–I; Figure S7A–H, Supporting Information).

In summary, our findings preliminarily indicate that Sono@NAT10 promotes macrophage reprogramming and suppresses the malignant behavior of CRC.

### 2.7 Sono@NAT10 Regulates Macrophage Reprogramming to Enhance CRC Immunotherapy Efficacy

**Figure 8:**
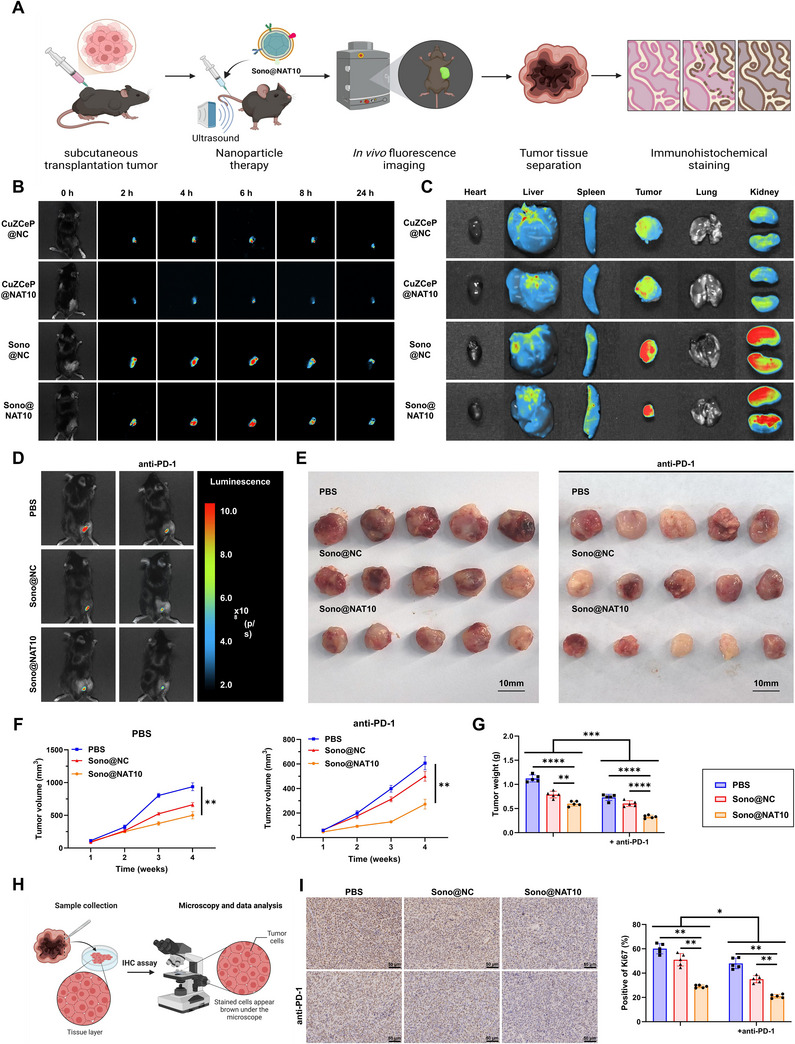
In vivo experimental results of nanomaterials. Note: A) Experimental workflow of Figure 8 (Created by Biorender); B,C) Fluorescence imaging to observe the distribution of fluorescence signals in major organs and tumor tissues in mice across groups; D) Tumor growth monitored via bioluminescence intensity at different time points, with one representative example shown for each group; E) Morphology of tumor tissues across groups; F) Tumor growth in mice across groups; G) Tumor tissue weights in each group; H,I) Immunohistochemical staining to evaluate Ki67 protein expression levels in tumor tissues of mice across groups (scale bar: 50 µm). Two‐way analysis of variance was used to analyze the data among the different time points and comparisons before and after treatment for each group. * indicates *p <* 0.05; ** indicates *p <* 0.01. Each group included five mice.

Previous in vitro experiments demonstrated that Sono@NAT10 enhances CRC immunotherapy efficacy by promoting macrophage reprogramming. To further confirm its therapeutic potential in vivo (**Figure** **8**A), we administered Sono@NAT10 via tail vein injection in mice. In vivo imaging demonstrated that both Sono@NC and Sono@NAT10 accumulated prominently at tumor sites, with Sono@NAT10 showing more sustained retention, which may be attributed to the targeting effect conferred by the macrophage membrane coating (Figure 8B,C).

Fluorescence staining of tumor tissues from the different groups showed that macrophage polarization in the Sono@NAT10 group was consistent with the in vitro findings. Specifically, M1 polarization was significantly enhanced, while M2 polarization was markedly suppressed (Figure S8A–B, Supporting Information). Consistently, Western blot results demonstrated that, relative to the PBS, CuZCeP@NC, and Sono@NC groups, tumor tissues from the Sono@NAT10 group exhibited significantly lower NAT10 and SRSF2 expression, accompanied by a notable upregulation of HDAC10 (Figure S8C, Supporting Information).

Building on the in vitro findings, we further investigated the effects of Sono@NC and Sono@NAT10 on immunotherapy efficacy in mice. Tumor bioluminescence imaging demonstrated that Sono@NAT10 significantly suppressed tumor growth compared to PBS, and its combination with anti‐PD‐1 (CD279) antibody achieved further tumor reduction (Figure 8D; Figure S9A, Supporting Information). Measurements of tumor volume and weight demonstrated a marked reduction in the Sono@NAT10 group relative to PBS and Sono@NC groups, with the greatest reduction observed under combined treatment with anti‐PD‐1 (CD279) antibody (Figure 8E–G; Figure S9B–D, Supporting Information).

IHC staining revealed a marked reduction of Ki67‐positive cells in the Sono@NAT10 group compared with PBS and Sono@NC groups, which was further enhanced by anti‐PD‐1 (CD279) antibody, indicating stronger tumor growth inhibition (Figure 8H,I; Figure S9E, Supporting Information).

Evaluation of liver function (ALT, AST) and kidney function (BUN, CREA) showed no significant group differences, indicating the absence of hepatic or renal toxicity (Figure S10A–D, Supporting Information). H&E staining of major organs post‐treatment did not reveal any pathological alterations, further confirming the biocompatibility of Sono@NAT10 (Figure S10E, Supporting Information).

These results demonstrate that Sono@NAT10 effectively enhances immunotherapy efficacy in vivo while maintaining excellent biocompatibility and safety.

## 3 Discussion

CRC remains a significant global health challenge.^[^ ^3^, ^4^ ^]^ Although surgery, chemotherapy, and radiotherapy have improved patient outcomes, their effectiveness in treating advanced or recurrent CRC remains limited.^[^ ^3^, ^5^, ^33^ ^]^ Immunotherapy has recently emerged as a therapeutic approach for CRC, largely through modulation of immune cell activity within the TME. Nevertheless, challenges such as immune tolerance, evasion, and incomplete regulation limit its efficacy. To address these issues, the bionic ultrasound‐sensitive nanorobot Sono@NAT10 developed in this study specifically targets and modulates macrophage functions, effectively enhancing immune responses within the TME, especially in synergy with immunotherapy. This approach not only offers new perspectives for CRC immunotherapy but also demonstrates both technical and practical innovations.

Previous studies on NAT10 mainly emphasized its roles in cellular senescence and ribosome biogenesis.^[^ ^34^ ^]^ More recent work has suggested a potential involvement of NAT10 in tumor progression.^[^ ^35^ ^]^ Distinct from prior investigations, this study demonstrates that NAT10 influences macrophage polarization through its phase‐separated condensates, providing a novel perspective on its function within the TME. Targeting these condensates highlights their critical role in macrophage polarization and identifies a promising therapeutic opportunity for cancer treatment.

Macrophage reprogramming from M2 to M1 is pivotal in enhancing tumor immunotherapy.^[^ ^36^, ^37^ ^]^ In this study, Sono@NAT10 achieves M1 macrophage polarization by reducing the acetylation level of SRSF2 protein and activating HDAC10, a mechanism rarely reported in prior research. Compared to conventional physical or chemical approaches for promoting macrophage polarization, the nanotechnology‐based approach provides a more precise and efficient strategy for modulating macrophage function, highlighting a significant advancement in cancer immunotherapy.

Although nanocarriers have been extensively studied in drug delivery systems, their application in immunotherapy remains at an early stage.^[^ ^38^, ^39^ ^]^ This study introduces a biomimetic, sono‐sensitive nanorobot designed to mimic biological membrane structures while enabling precise drug release triggered by the US. This innovative approach enhances targeting efficiency while minimizing systemic adverse effects, underscoring the potential of nanotechnology in reshaping the tumor immune microenvironment.

US‐triggered drug release systems offer a novel delivery strategy with distinct advantages over traditional temperature‐ or pH‐sensitive systems. Specifically, the US enables precise spatial and temporal control of drug release. In this study, the design of Sono@NAT10 facilitates the targeted release of siRNA under US stimulation, thereby enhancing therapeutic efficacy and minimizing off‐target effects on healthy tissues.

Using a murine CRC model, IF staining, FCM, and Western blot were applied to systematically assess the in vivo performance of the nanorobot. These integrated analyses confirmed the functionality of Sono@NAT10 and provided a strong experimental basis for future preclinical evaluation.

This study developed the Sono@NAT10 nanorobot, which reprograms macrophages from the M2 to M1 phenotype, significantly enhancing the efficacy of immunotherapy for CRC. By improving the performance of ICI such as anti‐PD‐1 (CD279) antibody, Sono@NAT10 offers a strategy to overcome adverse reactions and low response rates in patients. The nanorobot enables precise siRNA release with minimal toxicity to normal tissues, potentially reducing treatment‐associated side effects and improving quality of life. Future work will emphasize systematic safety and efficacy evaluation, including long‐term toxicological, immunogenicity (ADA, complement activation, cytokine profiling), and pharmacokinetic studies in large animal models. Early‐phase clinical trials (Phase 0) using advanced imaging will be designed to map in vivo behavior. In parallel, predictive biomarkers (e.g., M1/M2 ratio, NAT10 condensate dynamics, imaging signatures) will be established, and programmable, biodegradable, multi‐responsive carriers (ultrasound/pH/enzyme‐responsive) will be developed. Optimization of biodegradable sonosensitizers, low‐immunogenic engineered membranes, and siRNA co‐delivery systems with immunomodulators will be prioritized. Additionally, integration of ultrasound, photoacoustic, and MRI imaging into a theranostic platform will enable real‐time monitoring of nanorobot biodistribution and drug release. Closed‐loop ultrasound systems guided by imaging feedback, coupled with focused ultrasound techniques (e.g., HIFU) and portable devices, will further enhance spatial precision and clinical practicality. These directions aim to address material safety, targeting accuracy, controllable release, and patient heterogeneity, thereby laying a robust foundation for the clinical translation of Sono@NAT10 nanorobots.

Although this study achieved promising results in a murine model, several challenges must be addressed before clinical translation. First, the biological distribution, metabolism, and long‐term safety of the nanorobot in humans remain insufficiently studied. Nanomaterials may induce unpredictable immune responses or long‐term toxicity, particularly with repeated or prolonged use. The material‐related toxicity of Sono@NAT10 nanorobots arises from multiple factors, including the intrinsic toxicity of core components (carrier, sonosensitizer, membrane, siRNA), structural design parameters (size, surface properties, stability, responsiveness), functional mechanisms (ultrasound triggering and potential off‐target effects), and degradation or clearance pathways that may pose long‐term risks. Key concerns involve ROS generation by sonosensitizers in non‐target tissues, long‐term accumulation of nanocarriers in reticuloendothelial system organs, off‐target siRNA delivery, inappropriate ultrasound energy causing tissue injury, and immunogenicity of biomimetic membranes. For clinical translation, material design must prioritize precision, safety, and degradability. Key strategies include selecting biocompatible materials, optimizing biomimetic coatings to minimize immunogenicity, controlling particle size and surface properties, and developing highly responsive, low‐leakage ultrasound‐triggered systems. Comprehensive preclinical evaluations of acute, chronic, immune, and organ‐specific toxicity are essential, supported by advanced imaging for accurate pharmacokinetic and biodistribution analyses. Standardization of ultrasound treatment protocols will also be critical for toxicity control. A thorough understanding and careful management of material‐related risks are essential for the safe clinical translation of Sono@NAT10 nanorobots. Second, the controllability and precision of the US‐triggered release system require further validation in human settings, including the regulation of US energy, targeting performance, and stability in complex physiological environments. Furthermore, since the present work focused exclusively on CRC, the efficacy and adaptability of Sono@NAT10 in other tumor types remain uncertain.

Future studies should address these limitations through several key directions. Large‐scale preclinical investigations are required to evaluate the safety, pharmacokinetics, and biological effects of Sono@NAT10 across different tumor types and patient populations, providing a foundation for clinical translation. Optimization of nanorobot components and design will be essential to ensure both efficacy and safety in humans. Additionally, advancements in ultrasound technology are needed to improve the precision, controllability, and clinical feasibility of the drug release system. Personalized strategies should also be emphasized, with nanorobots tailored to the unique TME of individual patients to achieve more accurate therapeutic outcomes. By overcoming these challenges, Sono@NAT10 holds strong potential as an innovative approach for cancer treatment.

## 4 Conclusion

In conclusion, Sono@NAT10 blocks NAT10 phase‐separated condensates, reduces the acetylation level of SRSF2 protein, and reprograms macrophages from the M2 to M1 phenotype, thereby improving CRC immunotherapy efficacy (Graphic abstract). When combined with the immune checkpoint inhibitor anti‐PD‐1 (CD279) antibody, Sono@NAT10 effectively suppressed tumor growth and prolonged survival in mice. These findings present a promising immunotherapeutic strategy that employs sono‐sensitive nanorobots to precisely regulate macrophage polarization and strengthen antitumor immune responses.

## 5 Experimental Section

### Construction and Culture of In Vitro Cell Models

Mouse CRC cell lines CT26 (CRL‐2638; RRID: CVCL_7255) and CMT93 (CCL‐223; RRID: CVCL_4348) were purchased from ATCC (Manassas, VA, USA). CMT93 cells and RAW264.7 macrophages were cultured in Dulbecco's Modified Eagle Medium (DMEM; 11 965 092) enriched with 10% fetal bovine serum (FBS; 10099141C) and 1% penicillin–streptomycin (15 140 148). CT26 cells were maintained in RPMI‐1640 medium (A1049101) enriched with 10% FBS (12 484 028) and 1% penicillin–streptomycin (15 140 148). All culture reagents were purchased from Gibco (USA).

The 293T cell line (CRL‐3216; RRID: CVCL_0063, ATCC) was grown in DMEM containing 10% FBS, 10 µg mL^−1^ streptomycin, and 100 U mL^−1^ penicillin. All cultures were maintained at 37 °C in a humidified incubator with 5% CO_2_ (Heracell Vios 160i CR, Thermo Scientific, Germany). Cultures were routinely passaged at 80–90% confluence.^[^ ^40^ ^]^

Mycoplasma contamination was routinely screened using the MycoAlert Mycoplasma Detection Kit (LT07‐318, Lonza), and only negative‐tested cultures were used for experiments.

### Preparation of Fluorescently Labeled Tumor Cells

The pGL4.51 [luc2/CMV/Neo] luciferase reporter plasmid (Promega, USA) was used in this study (Figure S1A, Supporting Information). At ≈80% confluence, CT26 or CMT93 cells were transfected with the plasmid employing Lipofectamine 2000 (11 668 030, Gibco, USA) at a 1:2.5 ratio (Figure S1B, Supporting Information). After 24 h, the Geneticin antibiotic (10 131 035, ThermoFisher, USA) was added to select stably transfected cells, generating CT26‐Luc or CMT93‐Luc cells. The stably transfected cells in the logarithmic growth phase were seeded into 24‐well plates at the following densities: 2 × 10^6^, 1.5 × 10^6^, 1 × 10^6^, 0.8 × 10^6^, 0.6 × 10^6^, and 0.4 × 10^6^ cells per well, each cultured in 1 mL of medium. D‐luciferin (L9504, Sigma, USA, 30 mg mL^−1^) was added at 150 µL per well, and bioluminescence imaging was performed 5 min later using the IVIS Spectrum system (PerkinElmer) (Figure S1C, Supporting Information) to identify and confirm the labeled cells.

### Construction of a Subcutaneous Tumor Xenograft Model for CRC

Male specific pathogen‐free (SPF) C57BL/6J mice (6 weeks, 20–22 g) were obtained from Hunan SJA Laboratory Animal Co., Ltd. (Hunan, China). Animals were housed in an SPF facility under controlled humidity (60–65%), temperature (25 ± 2 °C), and a 12 h light/dark cycle, with free access to food and water. Following one week of acclimation, health status was assessed before experimentation. All animal procedures were approved by the Ethics Committee of The Affiliated Hospital of Jiangnan University and complied with the guidelines of the International Association for the Study of Pain

Stably transfected CMT93 (CCL‐223, ATCC, USA) cells (1 × 10^6^) were subcutaneously injected into the right flank of each mouse. Tumor development was monitored by bioluminescence imaging (IVIS Spectrum CT, PerkinElmer, USA). Tumor volume was calculated using the following formula: volume = (length × width^2^)/2.^[^ ^41^, ^42^ ^]^ Serum ALT (JN20465), AST (JN20681), BUN (JN55961), and CREA (JN7806) kits (Jining Bio, Shanghai, China) were used according to the manufacturer's protocols.^[^ ^43^ ^]^

Mice were randomly allocated into nine experimental groups (*n* = 5 each): 1) Tumor group (untreated tumor‐bearing mice); 2) PBS/Blank group (controls treated with PBS); 3) anti‐PD‐1 group (tumor‐bearing mice treated with Nivolumab); 4) CuZCeP@NC group (tumor‐bearing mice treated with CuZCeP@NC nanoparticles); 5) CuZCeP@NAT10 group (tumor‐bearing mice treated with CuZCeP@NAT10 nanoparticles); 6) Sono@NC group (tumor‐bearing mice treated with Sono@NC nanoparticles); 7) Sono@NAT10 group (tumor‐bearing mice treated with Sono@NAT10 nanoparticles); 8) Sono@NC + anti‐PD‐1 group (tumor‐bearing mice treated with Sono@NC nanoparticles and anti‐PD‐1 (CD279) antibody); and 9) Sono@NAT10 + anti‐PD‐1 group (tumor‐bearing mice treated with Sono@NAT10 nanoparticles and anti‐PD‐1 (CD279) antibody).Anti‐PD‐1 (CD279) (200 µg/mouse, clone RMP1‐14, Bio X Cell, USA) was administered via injection starting on day 6 after tumor cell implantation and given once every three days. Tumor‐bearing mice received tail vein injections of either 8 mg kg^−1^ PBS or nanoparticle solutions three times weekly, followed by US stimulation (1 W cm^−2^, 1 MHz, 0.45 MPa) for 3 min. Temperature changes were monitored to ensure stability within 12 h. The experiment concluded four weeks after tumor inoculation.

For intra‐tumoral imaging, excised tumor tissues were subjected to ex vivo imaging, fixed in 4% paraformaldehyde(PFA) for 24 h, and cryoprotected in 15% and 30% sucrose solutions sequentially. Samples were sectioned (20 µm), stained with DAPI (1 mg mL^−1^, 10 min), rinsed with PBS (pH 7.4), and observed utilizing confocal laser scanning microscopy (CLSM; LSM 700, Carl Zeiss, Germany).^[^ ^44^, ^45^ ^]^

### Hematoxylin and Eosin (H&E) Staining

Paraffin‐embedded tissue blocks were sectioned, deparaffinized with xylene, and rehydrated through a graded ethanol series (100%, 95%, and 70%) or distilled water. Sections were stained with hematoxylin (H8070, Solarbio, Beijing) for 5–10 min at room temperature (RT), rinsed in water. The sections were subsequently dehydrated in 95% ethanol, counterstained with eosin (G1100, Solarbio, Beijing, China) for 5–10 min, and passed through ascending ethanol concentrations (85%, 90%, 95%, and 100%). Finally, the sections were cleared with dichloromethane.

### Immunohistochemical (IHC) Staining for Ki67 Protein Expression

Subcutaneous tumor tissues were fixed overnight in 4% PFA, embedded in paraffin, and sectioned at 4 µm. After deparaffinization in xylene and rehydration through graded ethanol (100%, 95%, and 75%), antigen retrieval was performed by heating in 0.01 m citrate buffer for 15–20 min. Endogenous peroxidase activity was blocked with 3% H_2_O_2_ for 30 min at RT, followed by incubation in goat serum for 20 min. Sections were then incubated with anti‐Ki67 antibody (ab16667, 1:200, Abcam) for 1 h at RT and washed with PBS. A secondary antibody (ab150077, 1:1000, Abcam) was applied for 20 min at 37 °C, followed by streptavidin–peroxidase (SP) treatment for 30 min. Signals were visualized with DAB (P0202, Beyotime) for 5–10 min and counterstained with hematoxylin (C0107, Beyotime) for 2 min. Sections were dehydrated, cleared in xylene, and mounted with neutral resin. Slides were imaged under a light microscope, and five random high‐power fields were analyzed. The Ki67 index was determined as the percentage of positive nuclei among 100 counted cells per field.

### High‐Throughput Sequencing and Analysis of Macrophages

Tumor tissues and matched adjacent normal tissues were obtained from three CRC‐bearing mice. Macrophages were separated from these samples, and total RNA was extracted from six specimens using the Total RNA Isolation Kit (12 183 555, Invitrogen, USA). The RNA concentration and purity were measured with a UV spectrophotometer (BioSpectrometer basic, Eppendorf, USA), and integrity was verified by agarose gel electrophoresis. High‐quality RNA was reverse‐transcribed into cDNA for library preparation. Sequencing was performed on the Illumina NextSeq 500 platform, and base calling was used to convert raw image files into reads. Low‐quality sequences and adapters were removed using Cutadapt, producing clean reads. These clean reads were mapped to the mouse reference genome via HISAT2, and transcript levels were quantified to generate an expression matrix using R software.

Differentially expressed genes (DEGs) were determined with the “limma” package in R, applying |log_2_FC| > 1 and *p <* 0.01 as thresholds. Volcano plots and Venn diagrams were created through the Xiantao Academic platform (https://www.xiantaozi.com/), while heatmaps were generated through the Hiplot database (https://hiplot.com.cn/).

### Machine Learning for Key Gene Selection

The 375 differentially expressed genes identified were used as candidate features. LASSO‐logistic regression was performed using the “glmnet” package in R (v4.0‐2) to distinguish between tumor and adjacent normal tissue samples. Cross‐validation was applied to determine the optimal penalty parameter (λ) for feature selection. Subsequently, the SVM‐RFE (Support Vector Machine‐Recursive Feature Elimination) method was employed to further extract potential feature genes, and a random forest model was used to rank and evaluate the importance of the candidate genes.

### Preparation and Measurement of Proteomic Samples

Tumor tissues were collected from three mice in the PBS group and three in the Sono@NAT10 group. Macrophages were isolated and ground in liquid nitrogen using a mortar and pestle, and the resulting powder was transferred to 5‐mL centrifuge tubes. Cell lysis was performed on ice with an ultrasonic disruptor (SCIENTZ‐IID, Scientz, Ningbo, China) in extraction buffer containing phenol (100 206, Sigma‐Aldrich, USA), 10 mm dithiothreitol (DTT; R0861, Solarbio, Beijing), 1% protease inhibitor mixture (P6731, Solarbio, Beijing), and 2 mm ethylenediaminetetraacetic acid (EDTA; E1170, Solarbio, Beijing). An equal volume of Tris‐saturated phenol (pH 8.0; BIOFOUNT, Beijing) was added, vortexed for 4 min, and centrifuged at 5000 g for 10 min at 4 °C. The phenol phase was collected, and proteins were precipitated with 0.1 m ammonium sulfate in methanol (1:5, v/v; Sigma‐Aldrich, USA) and incubated overnight. After centrifugation, the protein pellets were washed once with cold methanol and three times with cold acetone, then dissolved in 8 m urea (Solarbio, Beijing). Protein concentrations were quantified using the bicinchoninic acid (BCA) assay kit (P0012, Beyotime Biotechnology, Shanghai, China).

### Protein Digestion, Peptide Labeling, Fractionation, and Nano‐Liquid Chromatography‐Mass Spectrometry/Mass Spectrometry (LC‐MS/MS) Detection

A total of 50 µg of protein from each sample was used for enzymatic digestion. DTT was added to a final concentration of 5 mm, and the mixture was incubated at 56 °C for 30 min. Iodoacetamide was then added to 11 mm and incubated at RT for 15 min. The urea concentration was diluted to below 2 m, and sequencing‐grade trypsin (25 200 056, Thermo Fisher, USA) was added at a protein‐to‐enzyme ratio of 50:1 (w/w) for overnight digestion at 37 °C. A second aliquot of trypsin was added at a ratio of 100:1 and digestion continued for 4 h.

Peptides were purified on a HyperSep C18 column, dried, re‐dissolved in 0.5 m triethylammonium bicarbonate (TEAB), and labeled with TMT reagents (Thermo Fisher, USA) for 2 h at RT. After equal pooling, desalting, and drying, samples were fractionated into 15 fractions using the Pierce High‐pH Reversed‐Phase Kit and reconstituted in 0.1% formic acid.

Two micrograms of peptides from each fraction were analyzed on an Easy‐nLC 1200 nano‐ultra‐performance liquid chromatography (nano‐UPLC) system (Thermo Fisher, USA). The samples were first loaded onto a Trap C18 column (100 µm × 20 mm, 5 µm) and further resolved on an analytical C18 column (75 µm × 150 mm, 3 µm) at a constant flow rate of 300 nL min^−1^. Mobile phases were 0.1% formic acid in water (A) and 0.1% formic acid in 95% acetonitrile (B), with a 90‐min gradient (2–8% B, 0–2 min; 8–28% B, 2–71 min; 28–40% B, 71–79 min; 40–100% B, 79–81 min; 100% B, 81–90 min). Peptides were analyzed on a Q‐Exactive HF‐X mass spectrometer with a spray voltage of 2.1 kV in positive ion mode. MS1 scans (m/z 350–1200) were acquired at 60 000 resolution (m/z 200), AGC target 3 × 10^6^, and maximum injection time (IT) 30 ms. MS2 scans were acquired at 15 000 resolution, AGC target 1 × 10^6^, 25 ms IT, using high‐energy collision dissociation (HCD; NCE 32) with a 2.0 Th isolation window.

### Database Search and Data Processing

LC–MS/MS data were processed utilizing MaxQuant (v1.5.2.8) for protein identification and quantification. The resulting MS/MS spectra were searched against the UniProt database (release 14.1, 2009; https://www.uniprot.org/) combined with a reversed decoy database. Trypsin/P was specified as the protease, allowing up to two missed cleavages. The precursor ion mass tolerance was set at 20 ppm for the first search and 5 ppm for the main search, while the tolerance for fragment ions was 0.02 Da. Data were filtered at a false discovery rate of ≤0.01 at both peptide and protein levels, and only peptides passing the score distribution threshold were retained. Differentially expressed proteins (DEPs) between the PBS and Sono@NAT10 groups were determined utilizing the “limma” package in R, with |log_2_FC| > 1 and *p *< 0.01 as selection criteria. The RNAInter database (http://rnainter.org/) was also used to identify proteins related to SRSF2.

### Protein–Protein Interaction (PPI) Network

The DEPs identified in macrophage samples were analyzed for PPI networks utilizing the STRING database (https://cn.string‐db.org/). The resulting interaction networks were visualized and mapped with Cytoscape software (v3.10.0).

### Preparation of CuZCeP@NAT10

Negative control (NC) siRNA (NC siRNA, 5ʹ‐UAUUCAGAGAGGUCCAUGCTT‐3ʹ) and NAT10 siRNA (NAT10 siRNA, 5ʹ‐GCACCACUGCUGAGAAUAATT‐3ʹ), both synthesized by Genechem (Shanghai, China), were dissolved in RNase‐free water (100 nm). The siRNA solution was mixed with 2.5 m 2‐methylimidazole (2‐MIM; M50850, Sigma‐Aldrich, USA) and stirred at 4 °C for 30 min. Subsequently, 0.5 m zinc acetate dihydrate (379 786, Sigma‐Aldrich) was slowly added at a weight ratio of 1:20 to form a turbid suspension, which was incubated for 20 min. The resulting nanoparticles, ZIF‐8@NC or ZIF‐8@NAT10, were harvested by centrifugation (10 000 g, 10 min) and washed three times with RNase‐free water. Next, 0.5 g of the synthesized ZIF‐8@NC or ZIF‐8@NAT10 was added to 40 mL of ethanol containing 0.24 g of copper nitrate trihydrate (1 mmol; 61 197, Sigma‐Aldrich). The mixture was stirred at RT for 1 h. The light blue product was obtained by centrifugation, washed three times with methanol, and vacuum‐dried at 60 °C to yield Cu‐ZIF‐8@NC (CuZ@NC) or Cu‐ZIF‐8@NAT10 (CuZ@NAT10).

Next, 20 µg of Chlorin e6 (Ce6; HY‐13594, MedChemExpress, USA) dissolved in methanol was mixed with 20 µg of CuZ@NC or CuZ@NAT10, also dissolved in methanol. After stirring at 4 °C for 4 h, the final nanocomposites, Cu‐ZIF‐8@NC@PFC@Ce6 (CuZCeP@NC) or Cu‐ZIF‐8@NAT10@PFC@Ce6 (CuZCeP@NAT10), were collected by centrifugation (6500 rpm, 30 min) and washed three times.

### Preparation of Sono@NAT10

Macrophage membranes were extracted from RAW 264.7 cells (TIB‐71, ATCC, USA). Cells were collected and resuspended at 2.5 × 10⁷ cells mL^−1^ in cold Tris‐Magnesium (TM) buffer (0.01 m Tris, 0.001 m MgCl_2_, pH 7.4). The suspension was disrupted by passing through an LF‐1 liposome extruder (Avestin, Canada) without a polycarbonate membrane 20 times. The lysate was adjusted with sucrose to a final concentration of 0.25 m and centrifuged at 2000 × g for 10 min at 4 °C to remove debris. The supernatant was further centrifuged at 3000 × g for 30 min at 4 °C to isolate cell membranes. After washing with 0.25 m sucrose/TM buffer and repeating centrifugation, purified macrophage membranes were collected. Protein content was quantified by BCA assay, yielding ≈0.28 mg membrane protein from 1 × 10⁸ cells.

Sono@NAT10 and Sono@NC were prepared by extruding macrophage membranes derived from 4 × 10^8^ cells with CuZCeP@NAT10 or CuZCeP@NC aqueous solutions (500 µg mL^−1^). The mixture was sequentially passed through polycarbonate membranes with pore sizes of 800, 400, and 200 nm. The nanomaterials were divided into four groups: 1) CuZCeP@NC group: ZIF‐8 framework modified with NC siRNA, Cu, Ce6, and PFC. 2) CuZCeP@NAT10 group: ZIF‐8 framework modified with NAT10 siRNA, Cu, Ce6, and PFC. 3) Sono@NC group: CuZCeP@NC nanoparticles encapsulated with macrophage membranes. 4) Sono@NAT10 group: CuZCeP@NAT10 nanoparticles encapsulated with macrophage membranes.

### Characterization of Nanorobots

The encapsulation efficiency of NAT10 siRNA in ZIF‐8 was evaluated by measuring the amount of free siRNA in the supernatant at different ZIF‐8/siRNA mass ratios (w/w) using a Nanodrop spectrophotometer(260 nm). siRNA stability was assessed via 2% agarose gel electrophoresis. The specific surface area and pore size distribution of ZIF‐8 and ZIF‐8@NC were determined using the Brunauer–Emmett–Teller and Barrett–Joyner–Halenda methods. X‐ray diffraction (XRD) patterns were obtained with a D8 Discover diffractometer (Bruker, Germany) over the 5–50° range. The copper and zinc contents in CuZ@NC and CuZ@NAT10 were quantified by inductively coupled plasma mass spectrometry (ICP‐MS; NexION 300×, PerkinElmer, USA). Functional groups were characterized by Fourier‐transform infrared spectroscopy (FT‐IR; NICOLET Nexus 870, USA).

Protein composition of Sono@NAT10 and Sono@NC was analyzed by SDS‐PAGE. Particle size, ζ‐potential, and morphology were determined by dynamic light scattering (DLS; Zetasizer Nano ZS90, Malvern, UK), transmission electron microscopy (TEM; JEM‐1400, JEOL, Japan), and scanning electron microscopy (SEM; JEOL 6700, Japan).

To evaluate size stability, Sono@NAT10 was suspended in equal volumes of deionized water, PBS (10 010 023, Gibco, USA), DMEM (11 965 092, Gibco, USA), and 10% FBS (A5670701, Gibco, USA), and incubated at 37 °C for 48 h. Particle size was monitored by DLS. The content of Cu and Ce6 was quantified by ICP‐MS and a Variskan Flash microplate reader (Thermo Fisher, USA), respectively. For cellular uptake analysis, Sono@NAT10 or Sono@NC was labeled with 5 µg mL^−1^ DiO (equivalent to 3 µg mL^−1^ Ce6) and used for detection.

Sono@NAT10 suspended in deoxygenated PBS was subjected to US stimulation (1 W cm^−2^, 1 MHz, 0.45 MPa) using a DJO‐2766 US therapeutic device (Chattanooga, USA). Oxygen release was monitored for 5 min with a dissolved oxygen analyzer (JPBJ‐608, Rex, China). US‐triggered drug release was evaluated by adding Sono@NAT10 (1 mL) to PBS‐Tween 80 solution (100:1, v/v) under pH 5.5 or 7.4, with or without US stimulation. Samples were placed in molecular weight cut‐off 1000 Da centrifuge tubes, and release was monitored at scheduled intervals (0, 0.25, 0.5, 1, 1.5, 2, 4, 8, 12, and 24 h). Ce6 absorption in the filtrate was quantified at 670 nm using a microplate reader, while Cu^2^⁺ levels were determined by ICP‐MS. For reactive oxygen species (ROS) detection, Singlet Oxygen Sensor Green (SOSG, 10 µm) was added to Sono@NAT10 (40 µg mL^−1^) and subjected to US stimulation for 3 min under normoxic or hypoxic conditions. Fluorescence changes in SOSG were measured using a microplate reader, with free Ce6 and non‐O_2_‐loaded Sono@NAT10 as controls.

### Hemocompatibility Test

To evaluate the in vitro hemocompatibility of the nanomaterials, the hemolysis rate was determined after 1 h of contact with blood at 37 °C. Briefly, each group of nanomaterials was dispersed in 7 mL of 0.9% sodium chloride (NaCl) solution, followed by the addition of 0.2 mL of fresh anticoagulated blood, and then incubated at 37 °C for 1 h. After centrifugation (3000 rpm, 5 min, RT), the supernatant absorbance at 545 nm was measured (A). For controls, a mixture of 0.2 mL anticoagulated blood and 7 mL 0.9% NaCl solution served as the negative control, and a mixture of 0.2 mL anticoagulated blood and 7 mL H_2_O served as the positive control. The optical density (OD) values at 545 nm for the negative control (A^−^) and positive control (A^+^) were recorded. The hemolysis rate was calculated using the following formula:

(1) $$\mathrm{Hemolysis}\;\mathrm{rate}\left(\%\right)=\left(A-A^{-}\right)/\left(A^{+}-A^{-}\right)\times 100\%$$

### Construction and Transfection of Overexpression Vectors

A lentiviral vector encoding SRSF2 was generated by Genechem (Shanghai, China) using the pLenti‐GFP backbone. Recombinant plasmids were co‐transfected with packaging vectors into 293T cells (80–90% confluence) with Lipofectamine 2000 (11 668 500, Invitrogen, USA). Viral supernatants were collected after 48 h, filtered, and concentrated by centrifugation.

For infection, logarithmically growing cells were seeded in 6‐well plates (5 × 10⁴ cells mL^−1^, 2 mL well^−1^) and transduced with lentivirus (MOI = 10, 1 × 10⁸ TU mL^−1^) in the presence of 8 µg mL^−1^ polybrene (428 175, Sigma‐Aldrich, USA). After 48 h, stable cell lines were selected with 2 µg mL^−1^ puromycin (UC0E03, Sigma‐Aldrich, USA) for two weeks. For overexpression models, cells were further screened using ampicillin (171 254, Sigma‐Aldrich, USA) for two weeks.

### Cell Co‐Culture

Murine macrophage RAW 264.7 cells were polarized into M2 macrophages by supplementing the culture medium with 20 ng mL^−1^ IL‐10 (HY‐P7098A, MedChemExpress, USA).^[^ ^46^ ^]^ The in vitro M2 macrophage model was seeded into the upper chambers of a Transwell co‐culture system (CLS3412, Corning, USA) and placed in blank 6‐well plates. After experimental pre‐treatment, the medium was removed, wells were rinsed twice with PBS, and the Transwell chambers were transferred to plates seeded with CRC cells (CT26 or CMT93). M2 macrophages and CRC cells were co‐cultured at a ratio of 2:1 for 36 h. The supernatants and CRC cells from the lower chambers were collected for further analysis.^[^ ^47^, ^48^, ^49^, ^50^ ^]^ For nanoparticle treatment, 40 µg mL^−1^ of nanoparticles were applied to the M2 macrophage culture.

Co‐culture groups were organized as follows: 1) PBS group: Blank control group treated with PBS solution. 2) CuZCeP@NC group: Co‐culture of M2 macrophages treated with CuZCeP@NC particles and CRC cells. 3) CuZCeP@NAT10 group: Co‐culture of M2 macrophages treated with CuZCeP@NAT10 particles and CRC cells. 4) Sono@NC group: Co‐culture of M2 macrophages treated with Sono@NC particles and CRC cells. 5) Sono@NAT10 group: Co‐culture of M2 macrophages treated with Sono@NAT10 particles and CRC cells.

### Uptake of Sono@NAT10 by Cancer Cells

To examine cellular uptake of nanomaterials, RAW 264.7 cells (1 × 10^5^ cells per well) were seeded in 24‐well plates and treated with various nanomaterials (40 µg mL^−1^) for 12 h. The cells were then digested with trypsin, and the intracellular signal intensity of Ce6 at different time points was analyzed using a flow cytometer (FACSVerse, USA).

For confocal imaging, CT26 or CMT93 cells (1 × 10^5^ cells per dish) were seeded in 35‐mm glass‐bottom dishes and co‐cultured with different nanomaterial groups (40 µg mL^−1^, excitation = 410 nm, emission = 670 nm) for 6 h. The cells were fixed with 4% PFA, stained with DAPI (excitation = 350 nm, emission = 460 nm), and imaged using a CLSM (FV3000, Olympus, Japan).

For intracellular colocalization studies, CT26 or CMT93 cells were exposed to DiO‐labeled nanomaterials (5 µg mL^−1^ DiO; Ex 484 nm/Em 501 nm) for 6 h. After US stimulation (1 W cm^−^^2^, 1 MHz, 3 min), cells were fixed with 4% PFA, counterstained with DAPI, and imaged with CLSM.

### NAT10 Protein Purification and In Vitro Phase Separation Assay

Full‐length NAT10 cDNA was cloned into the pET‐28a vector containing dual tags of polyhistidine (His) and green fluorescent protein/Flag (GFP/Flag). After sequence validation, the construct was transformed into *Escherichia coli* BL‐21 (DE3) cells (C600003, Invitrogen, USA). Bacterial cultures were grown in Luria–Bertani (LB) medium and induced with 1 mm isopropyl β‐D‐1‐thiogalactopyranoside (IPTG; 15 529 019, Invitrogen, USA) at 19 °C, 200 rpm for 16 h. The recombinant His‐tagged NAT10 fusion protein was purified using a His‐tag protein purification kit (P2229S, Beyotime, China). All procedures were conducted by Genechem (Shanghai, China).

The full‐length NAT10 cDNA plasmid with His and GFP/Flag dual tags was also transfected into RAW 264.7 cells using Lipofectamine 3000 (L3000150, Invitrogen, USA). The RAW 264.7 cells were concurrently treated with nanomaterials. To assess the phase separation capacity of the NAT10 protein, cell lysates were diluted to the desired concentration in phase separation buffer containing 12.5 mm HEPES (pH 7.5; 15 630 130, Invitrogen, USA) and 0.5 mm DTT (D1532, Invitrogen, USA). Additionally, purified NAT10 protein at varying concentrations was mixed with buffers containing 10% PEG8000 (P5413, Sigma‐Aldrich, USA), 1,6‐hexanediol (240 117, Sigma‐Aldrich, USA), and NaCl (37.5–500 mm), or distilled water. Biomolecular condensates were visualized by CLSM.

### Real‐Time Imaging and Fluorescence Recovery after Photobleaching (FRAP)

RAW 264.7 cells were seeded in confocal dishes and transfected with GFP‐NAT10 plasmids for 24 h. The cells were incubated with DAPI, washed three times with fresh culture medium, and imaged utilizing a CLSM (TCS SP8, Leica, Germany). For FRAP analysis, NAT10 protein condensates were photobleached with a 488 nm laser at 50% power for 3 s. Recovery of fluorescence was monitored by capturing images every 5 s for a total of 150 s. The fluorescence intensity of bleached spots was normalized to pre‐bleaching levels.

### Co‐Immunoprecipitation (Co‐IP) to Study NAT10 and SRSF2 Interaction

Cells were lysed in NP‐40 buffer (P0013F, Beyotime, China). A portion of lysate (40 µg) was used as input, and the remaining protein was adjusted to 1 mg mL^−1^ and aliquoted into three tubes (1 mL each). The samples were incubated overnight at 4 °C with anti‐NAT10 antibody (10 µg; MA5‐42504, Invitrogen), anti‐SRSF2 antibody (10 µg; PA5‐78164, Invitrogen), or IgG control antibody (10 µg). Protein A/G agarose beads (sc‐2003, Santa Cruz, USA) were added and rotated for 4 h at 4 °C. After three washes with pre‐chilled TBS, bound proteins were eluted and analyzed by Western blot.

### Assessment of SRSF2 Acetylation

RAW 264.7 cells were transfected with either Myc‐tagged SRSF2 or the acetylation‐deficient mutant Myc‐SRSF2‐K52R (pcDNA3.1, Genechem, China). Cells were treated with PBS or Sono@NAT10 for 48 h, followed by 10 µm MG132 (HY‐13259, MedChemExpress, USA) for 8 h. Lysates were immunoprecipitated with anti‐Myc antibody (PA5‐85185, Invitrogen) at 4 °C for 24 h. The immunocomplexes were captured with Protein A‐Sepharose beads (9863, Cell Signaling Technology, USA) and washed extensively. Acetylation levels were examined by immunoblotting (IB) using anti‐acetyl‐lysine (Ac‐K) antibody (9681T, Cell Signaling Technology, USA).

### Determination of SRSF2 Protein Stability

To evaluate SRSF2 stability, macrophages were treated with PBS or Sono@NAT10 and exposed to cycloheximide (CHX, 3 µg L^−1^; M4879, AbMole, USA). Cells were lysed in RIPA buffer (P0013B, Beyotime, China), and lysates were incubated with PNGase F (G5166‐50UN, Sigma‐Aldrich, USA), followed by centrifugation at 12 000 rpm. Proteins were further treated with MG132 and lysed in RIPA buffer supplemented with 0.1% SDS. IB was performed to determine SRSF2 expression. Band intensities were quantified with ImageJ software and normalized to β‐actin. Protein samples collected at 0, 0.5, 1, and 1.5 h were used to construct the SRSF2 degradation curve.

### Enzyme‐Linked Immunosorbent Assay (ELISA)

Cytokine concentrations of TNF‐α (900‐T54), IL‐6 (900‐M50), TGF‐β (BMS808‐4), and IL‐10 (900‐M53) were quantified using ELISA kits (Invitrogen, USA). Absorbance was recorded at 450 nm with a microplate reader.

### CCK‐8 Assay for CRC Cell Viability

Cell viability was assessed with a CCK‐8 kit (C0037, Beyotime, Shanghai). CRC cells in logarithmic growth were resuspended at 5 × 10⁴ cells mL^−1^ in DMEM‐H supplemented with 10% FBS, and 100 µL per well was seeded into 96‐well plates. After 48 h of incubation, the medium was replaced with 10 µL CCK‐8 solution per well. Plates were incubated at 37 °C for 2 h, and absorbance at 450 nm was measured utilizing a Multiskan FC microplate reader (51 119 080, Thermo Fisher, USA). Each condition was tested in triplicate, and mean values were calculated.

### EdU Assay for CRC Cell Proliferation

Cells were seeded into 24‐well plates and incubated with EdU (C10310‐2, Guangzhou RiboBio Co., Ltd, Guangzhou) at a final concentration of 10 µmol L^−1^ for 2 h. After incubation, cells were fixed in 4% PFA for 15 min at RT, washed with PBS containing 3% bovine serum albumin (BSA), and permeabilized with 0.5% Triton X‐100 for 20 min. After two washes with PBS/BSA, 100 µL of staining solution was added, followed by incubation in the dark for 30 min. Nuclei were counterstained with DAPI for 5 min. Fluorescence images were acquired using a microscope (BX63, Olympus, Japan) from 6–10 random fields. The EdU labeling rate was calculated as: EdU labeling rate (%) = Positive cells/(Positive + Negative cells) × 100%. Each experiment was performed in triplicate.

### Wound Healing Assay

Cells were plated in 6‐well dishes and cultured to 70–90% confluence. A linear scratch was introduced with a 200 µL pipette tip, and floating cells were removed by PBS wash. Fresh medium was added, and cells were maintained at 37 °C with 5% CO_2_. Images of wound gaps were taken at 0 and 24 h using an inverted microscope (CKX53, Olympus, Japan). The wound closure was quantified by measuring the migration distance with ImageJ software.

### Transwell Assay for Evaluating CRC Cell Invasion

The upper chamber (8 µm pore size; 24‐well format) was coated with extracellular matrix (ECM) gel (E1270, Sigma‐Aldrich, Germany) and incubated at 37 °C for 30 min. Cells (2 × 10⁴ well^−1^), collected 48 h after transfection and suspended in serum‐free medium, were seeded into the upper chamber, while the lower chamber contained 20% FBS‐conditioned medium (800 µL). After 24 h at 37 °C, cells were washed with PBS, fixed in formaldehyde (10 min), and stained with 0.1% crystal violet (30 min). Non‐invading cells were removed, and invaded cells were imaged (CKX53, Olympus, Japan) and quantified with ImageJ.

### Flow Cytometry(FCM) for CRC Cell Apoptosis

Cells were digested with 0.25% trypsin (without EDTA) and collected into FCM tubes. After centrifugation, the supernatant was discarded, and the cells were washed three times with cold PBS, followed by centrifugation and removal of the supernatant. Staining was performed using an Annexin‐V‐FITC apoptosis detection kit (K201‐100, Biovision, USA). The staining solution was prepared by mixing Annexin‐V‐FITC, propidium iodide (PI), and HEPES buffer (1:2:50). A total of 1 × 10⁶ cells were resuspended in 100 µL staining solution, incubated at RT for 15 min, and then diluted with 1 mL HEPES buffer. Apoptotic cells were analyzed on a flow cytometer with 488 nm excitation, and FITC and PI signals were detected at 525 and 620 nm, respectively. Each experiment was performed in triplicate.

### FCM for Detecting Macrophage Content

Macrophages from the co‐culture system or tumor tissues were collected by centrifugation and digested with StemPro Accutase (A1110501, Gibco, USA) at 4 °C for 30 min. After washing twice with PBS, single‐cell suspensions were resuspended in 100 µL PBS and stained with the following antibodies: PE‐anti‐F4/80 (12‐4801‐82, rat anti‐mouse), Alexa Fluor 488‐anti‐CD11b (53‐0112‐82, rat anti‐mouse), APC‐anti‐CD86 (17‐0862‐82, rat anti‐mouse), and PE‐Cy7‐CD206 (25‐2061‐82, rat anti‐mouse) (all from Invitrogen, Thermo Fisher Scientific, USA). Following staining and three PBS washes, samples were analyzed utilizing a flow cytometer (Beckman, USA).

### RT‐qPCR for Relative Gene Expression Analysis

Total RNA was extracted from tissues or cells with Trizol reagent (15 596 026, Invitrogen, USA). RNA concentration and purity were assessed by NanoDrop LITE spectrophotometer (ND‐LITE‐PR, Thermo Scientific, Germany) based on the 260/280 nm absorbance ratio. cDNA was synthesized with the PrimeScript RT reagent kit with gDNA Eraser (RR047Q, TaKaRa, Japan). Quantitative PCR was performed on an ABI PRISM 7500 system with SYBR Green Master Mix (4 364 344, Applied Biosystems, USA).

Primers for each gene (Table S1, Supporting Information) were synthesized by TaKaRa, with GAPDH serving as the reference gene. Relative gene expression was calculated using the 2^−ΔΔCt^ method. All reactions were performed in triplicate.

### Western Blot Analysis

Cells or tissues were lysed in RIPA buffer containing protease inhibitors (P0013B, Beyotime, China), and protein concentrations were determined using a BCA assay kit (P0012, Beyotime, China). Equal amounts of protein were resolved by 10% SDS‐PAGE and transferred onto PVDF membranes (FFP39, Beyotime). Membranes were blocked with 5% BSA (ST023, Beyotime) for 2 h at RT, followed by incubation with primary antibodies (Table S2, Supporting Information) for 1 h. After washing, HRP‐conjugated goat anti‐rabbit IgG (ab6721, 1:2000, Abcam, UK) was applied for 1 h. Protein bands were visualized using the Pierce ECL substrate (32 209, Thermo Scientific, Germany) and imaged with a Bio‐Rad system. Band intensities were quantified using ImageJ, with β‐actin as the loading control. All experiments were performed in triplicate.

### Immunofluorescence (IF) Staining

Cells were seeded onto coverslips in 12‐well plates one day before staining. After attachment, coverslips were washed with DPBS (14 040 133, Gibco, USA) and fixed in 4% PFA (I28800, Thermo Scientific, USA) for 1 h, followed by washing with DPBS containing 0.05% Tween‐20 (655 204, Sigma‐Aldrich, USA). Cell membranes were permeabilized with 0.1% Triton X‐100 (HFH10, Invitrogen, USA) for 3 min, then blocked with DPBS containing 5% goat serum (16 210 072, Gibco, USA) and 0.3 m glycine (50 046, Sigma‐Aldrich, USA) for 1 h. Samples were incubated with primary antibodies (Table S2, Supporting Information) overnight at 4 °C. For double staining, an additional blocking step with 5% goat serum for 2 h was performed before applying the second antibody. The next day, samples were washed and incubated with fluorophore‐conjugated secondary antibodies for 1 h at RT. Nuclei were counterstained with DAPI (C1002, Beyotime, China) for 5 min, and slides were mounted with antifade medium. Images were acquired using a fluorescence microscope (FV‐1000/ES, Olympus, Japan). Quantification was performed by calculating the fluorescence‐positive area under a 40× objective in six random fields per group, and the average value was used for analysis.

### Statistical Software and Data Analysis

All analyses were conducted using R (v4.2.1) with RStudio (v2022.12.0‐353). Perl (v5.30.0) was used for file preprocessing, and figures were generated with GraphPad Prism (v8.0). Quantitative results were presented as mean ± SD. For data preprocessing, multiple imputation (mice R package) was applied for missing continuous variables and excluded missing categorical variables. Outliers were identified and removed using Tukey's fences method (threshold: 1.5 × IQR). Group comparisons were assessed by independent‐samples *t‐*test or one‐way analysis of variance (ANOVA), and time‐course data by two‐way ANOVA. Bonferroni correction was applied for multiple testing, with significance set at *p* < 0.05.

### Ethical Statement

All animal procedures were approved by the Ethics Committee of The Affiliated Hospital of Jiangnan University and complied with the guidelines of the International Association for the Study of Pain.

## Conflict of Interest

The authors declare no conflict of interest.

## Author Contributions

J.Z., S.J., Y.Z., and K.G. conceived and designed the study. J.Z., B.X., R.H., B.Y., and P.S. performed the experiments. Y.X., X.Z., D.Q., and G.W. analyzed the data. J.Z., S.J., Y.Z., and K.G. wrote the manuscript. All authors reviewed and approved the final version of the manuscript.

## Supporting information



Supporting Information

## Data Availability

The data that support the findings of this study are available from the corresponding author upon reasonable request.
