# Supplementary material for: Reprogramming Tumor‐Associated Macrophages via Targeted NAT10 Inhibition to Enhance Colorectal Cancer Immunotherapy
Source: Adv Sci (Weinh). 2025 Oct 27;13(2):e10854. doi: 10.1002/advs.202510854 (PMC12786336; doi:10.1002/advs.202510854)
Supplement: Supplementary file 1 — Supporting Information [file ADVS-13-e10854-s001.docx]

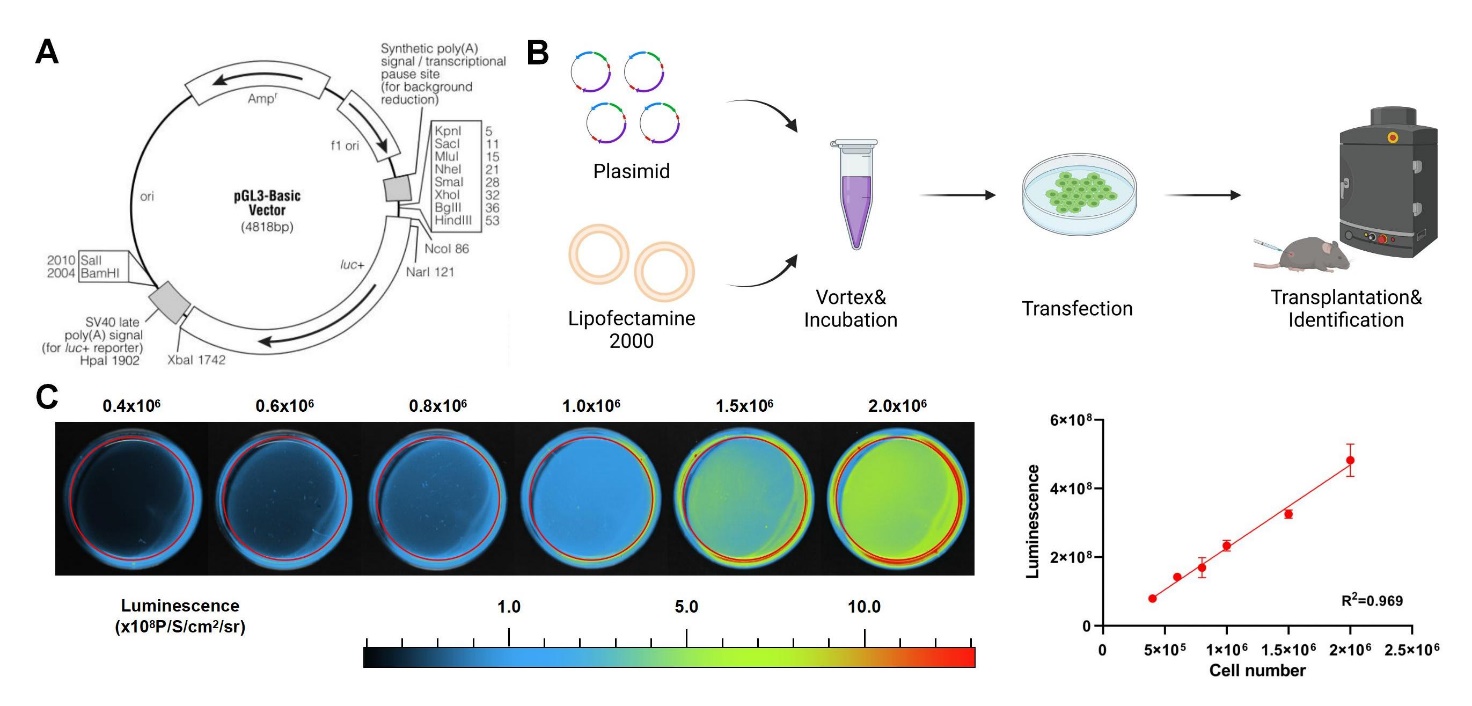


**Figure S1. Preparation and characterization of luciferase-labeled macrophages.**

Note: (A) Schematic representation of the pGL4.51 [luc2/CMV/Neo] plasmid structure; (B) Transfection workflow of the luciferase reporter plasmid; (C) Correlation analysis between fluorescence intensity and cell count in transfected cells under different cell densities. All cell experiments were repeated three times.


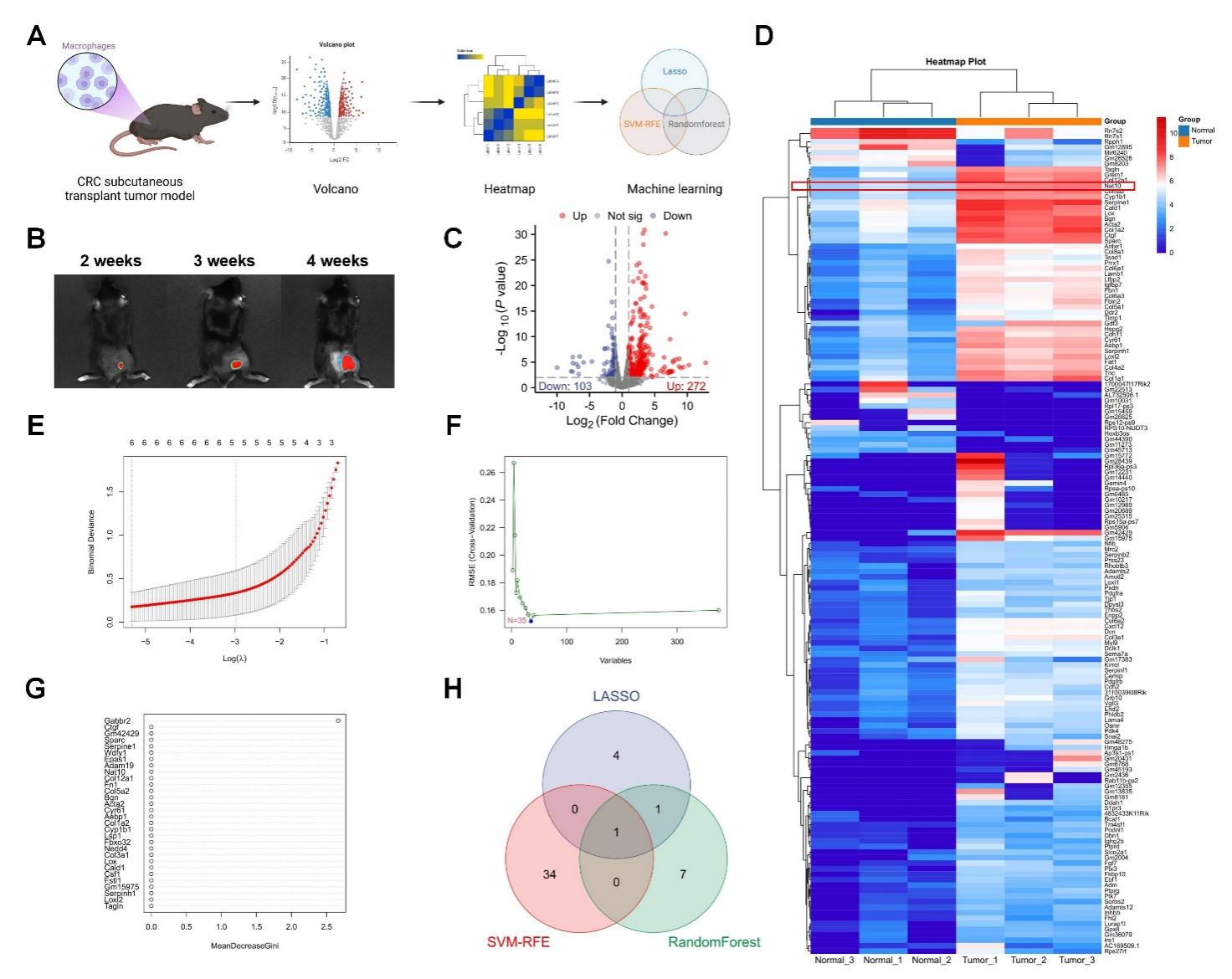


**Figure S2. A machine learning algorithm for identifying key factors in CRC macrophages.**

Note: (A) Workflow diagram of high-throughput sequencing analysis (Created by Biorender); (B) Validation results of CRC mouse model construction; (C) Volcano plot of differentially expressed mRNAs between tumor tissues and adjacent normal tissues in three successfully modeled mice based on high-throughput sequencing data; (D) Heatmap showing differential gene expression in macrophage samples from tumor and adjacent normal tissues; (E) LASSO coefficient selection plot; (F) Random forest algorithm results; (G) SVM-RFE analysis results; (H) Venn diagram showing the intersection of key factors identified by LASSO regression, random forest algorithm, and SVM-RFE analysis. In the volcano plot, blue dots represent significantly downregulated mRNAs in tumor tissues, red dots represent significantly upregulated mRNAs in tumor tissues, and gray dots represent mRNAs with no significant difference. Normal (N=3) and Tumor (N=3).


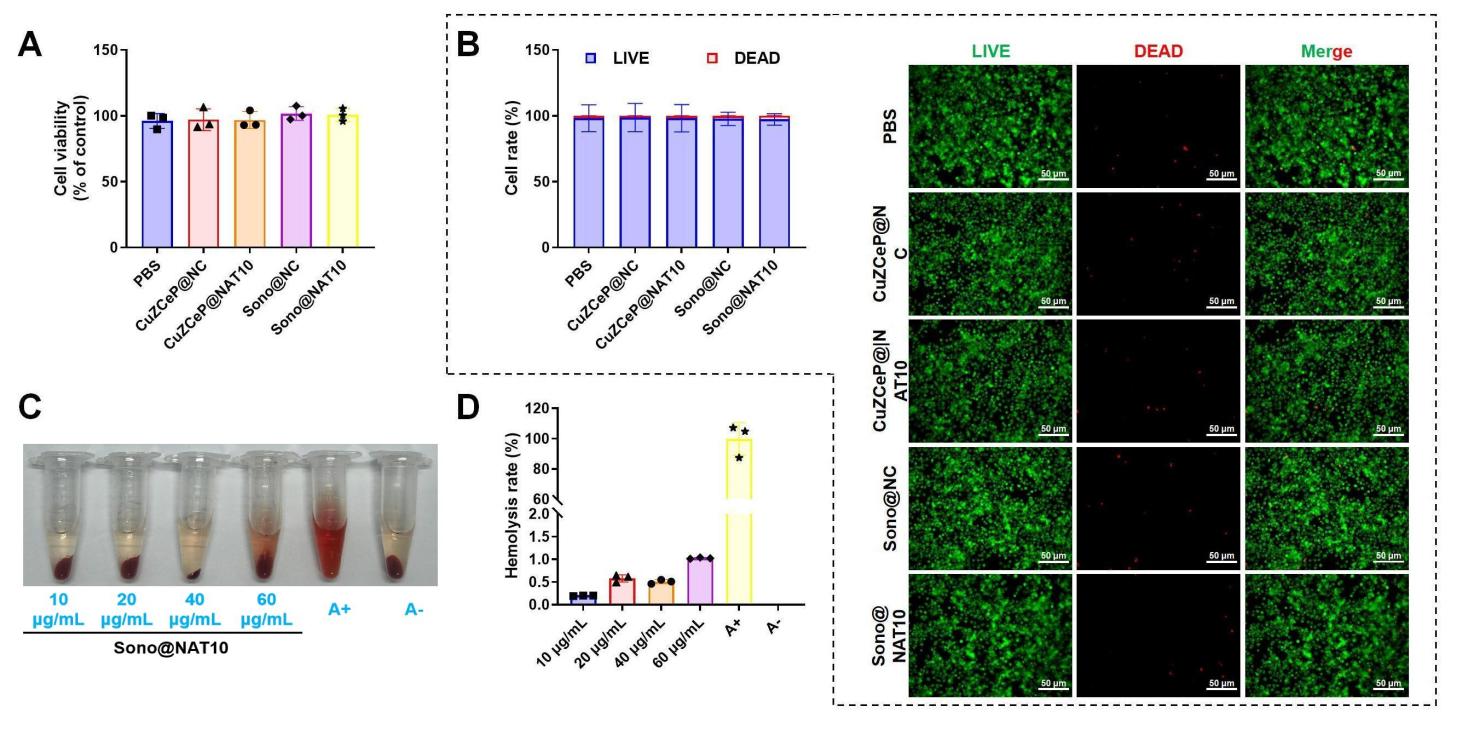


**Figure S3. Assessment of cellular viability and hemolysis rate induced by nanoparticles.**

Note: (A) Evaluation of cell proliferation using the CCK-8 assay; (B) Live/dead staining of cells in different nanoparticle treatment groups, with green staining indicating live cells and red staining indicating dead cells. The proportion of live and dead cells was calculated, scale bar = 50 μm; (C-D) Hemolysis rates induced by nanoparticles at different concentrations, where A^-^ and A^+^ represent the negative control (0.9% NaCl solution) and the positive control (H_2_O), respectively. Comparisons among different groups were performed using one-way ANOVA, *** *p* < 0.001. All cellular experiments were repeated three times.


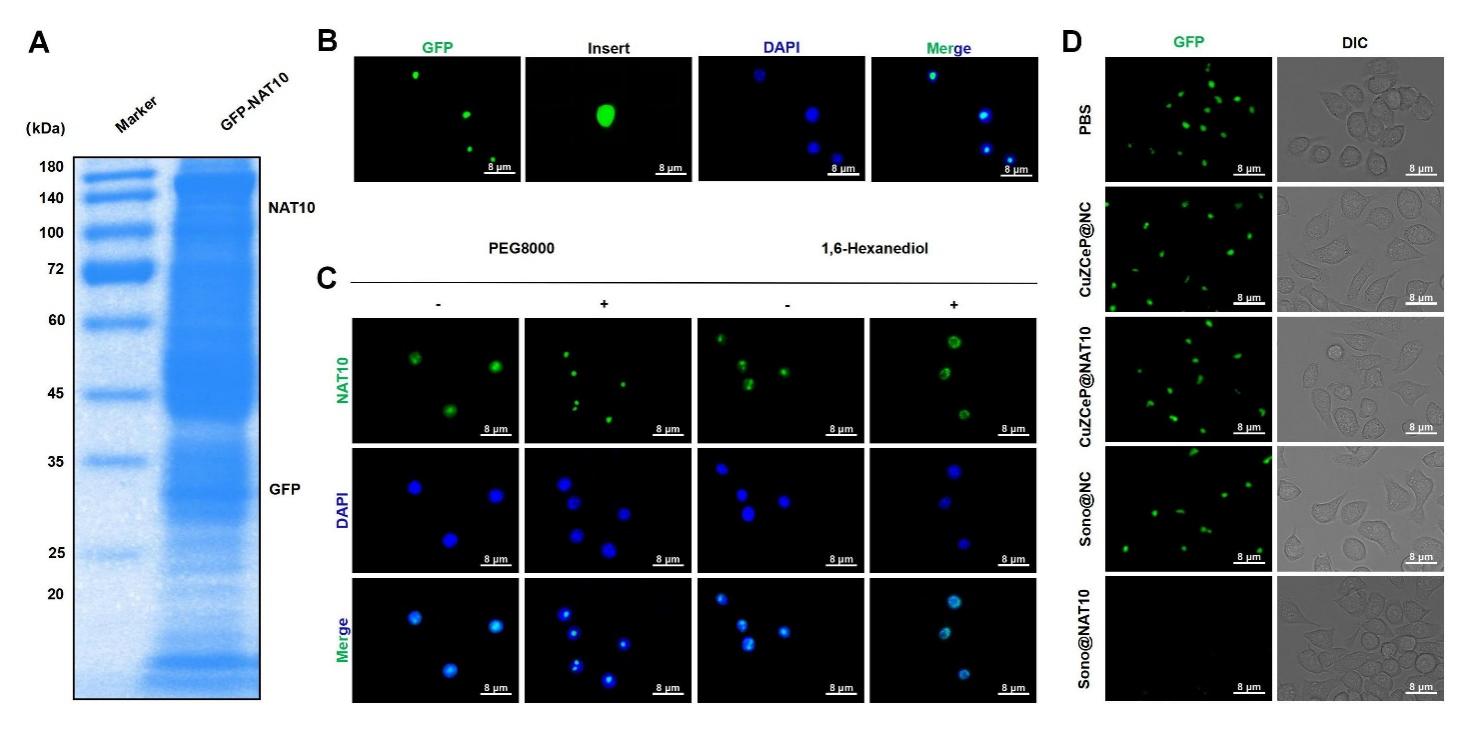


**Figure S4. Purification and phase separation observation of NAT10 protein.**

Note: (A) Results of NAT10 protein purification; (B) Formation of NAT10 condensates in macrophages, scale bar = 8 μm; (C) Observation of NAT10 phase-separated condensates in macrophages after interference with PEG8000 or 1,6-hexanediol, scale bar = 8 μm; (D) Formation of NAT10 condensates in macrophages treated with different nanoparticle groups, scale bar = 8 μm. All cellular experiments were repeated three times.


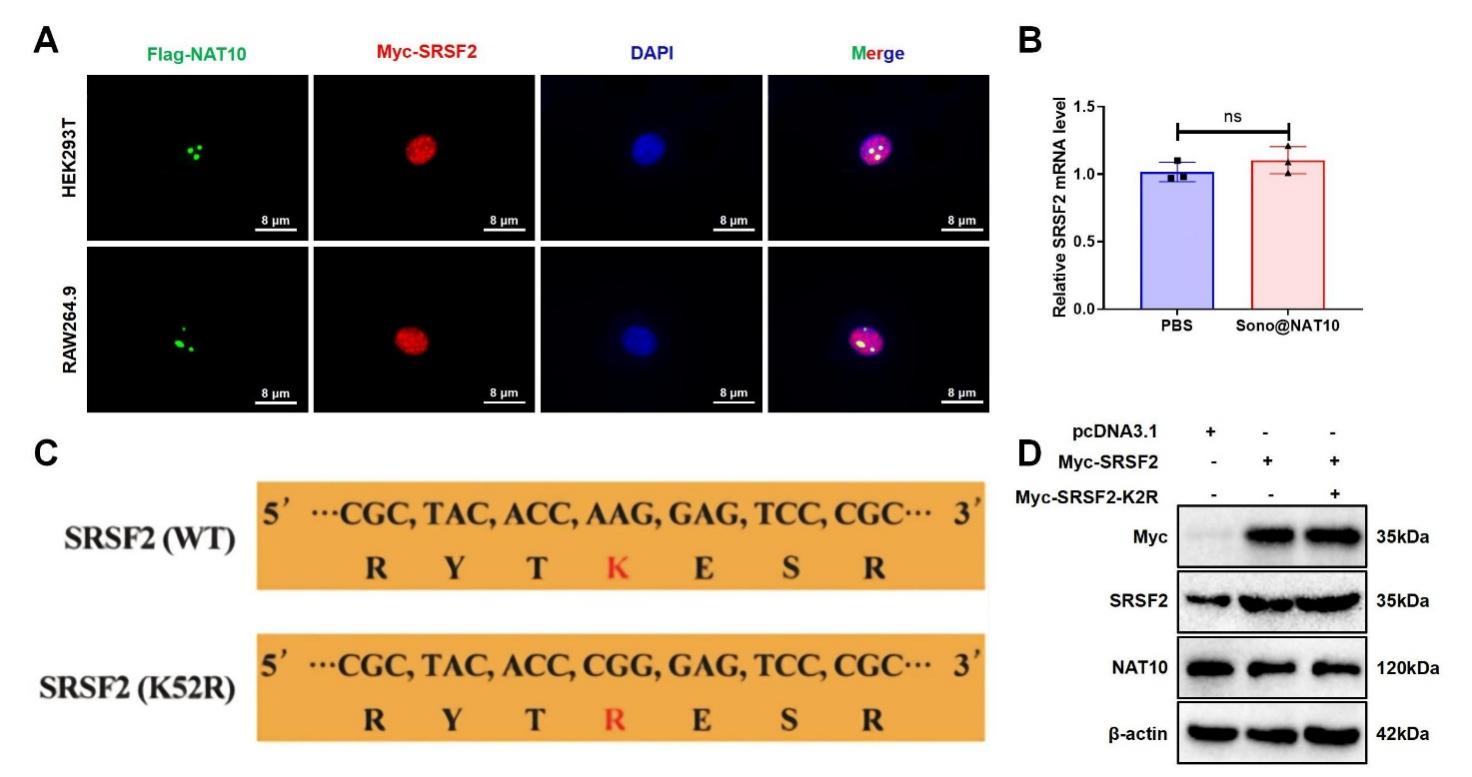


**Figure S5. Colocalization and interaction between NAT10 and SRSF2.**

Note: (A) Fluorescence imaging results showing colocalization of NAT10 and SRSF2, scale bar = 8 μm; (B) Effect of PBS or Sono@NAT10 treatment on SRSF2 mRNA levels; (C) Sequence differences between SRSF2 and its mutant form SRSF2-K52R (non-acetylatable variant); (D) Stability of vectors expressing SRSF2 and SRSF2-K52R. Comparison of the two groups was conducted using an independent samples t-test. All cellular experiments were repeated three times.


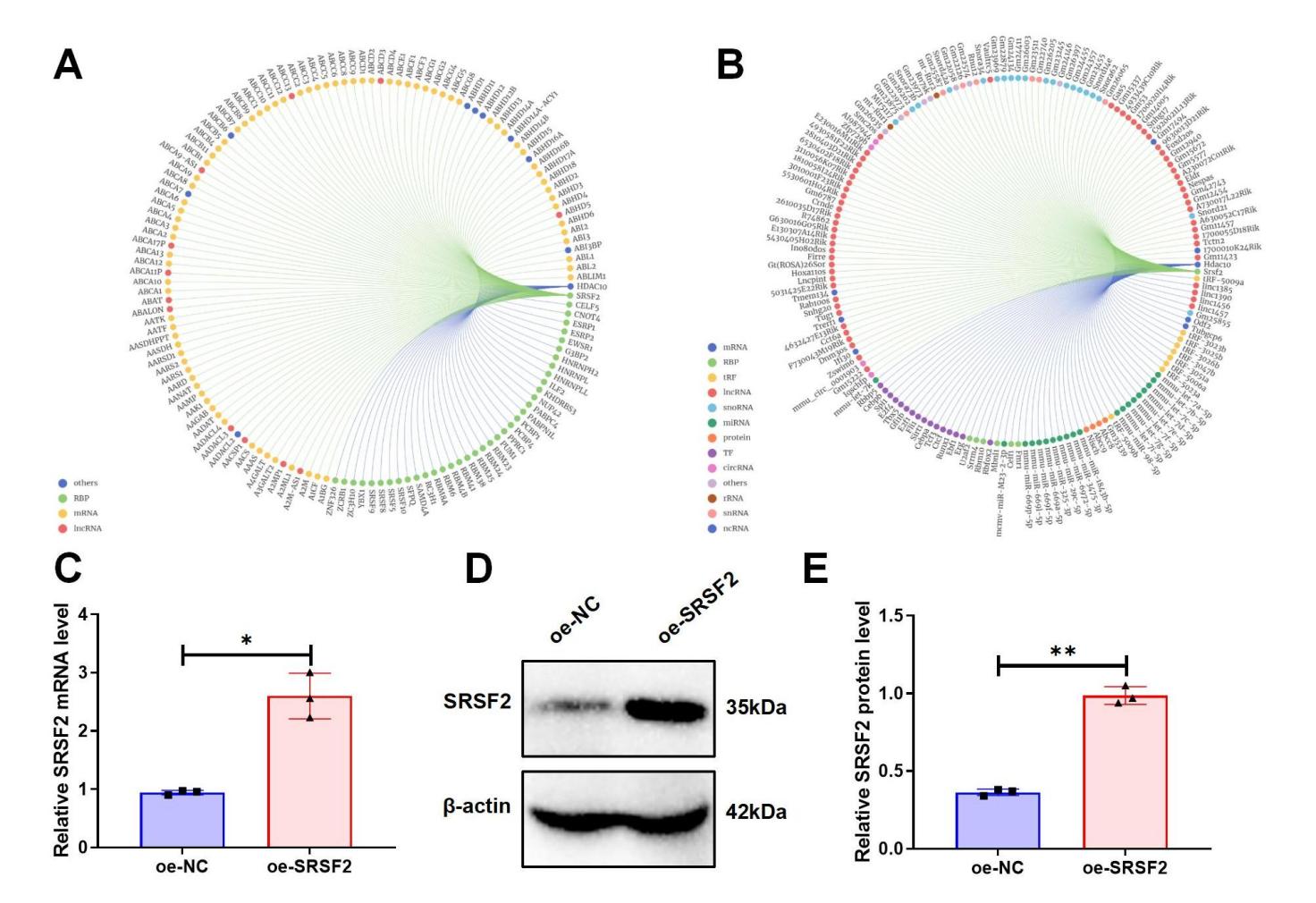


**Figure S6. Database analysis and lentiviral transfection results.**

Note: (A-B) Correlation analysis between SRSF2 and HDAC10 based on RNAInter database results; (C) RT-qPCR analysis of lentiviral transfection efficiency; (D-E) Western blot analysis of lentiviral transfection efficiency. All cellular experiments were repeated at least three times, with values presented as mean ± standard deviation. * indicates *p <* 0.05; ** indicates *p <* 0.01.


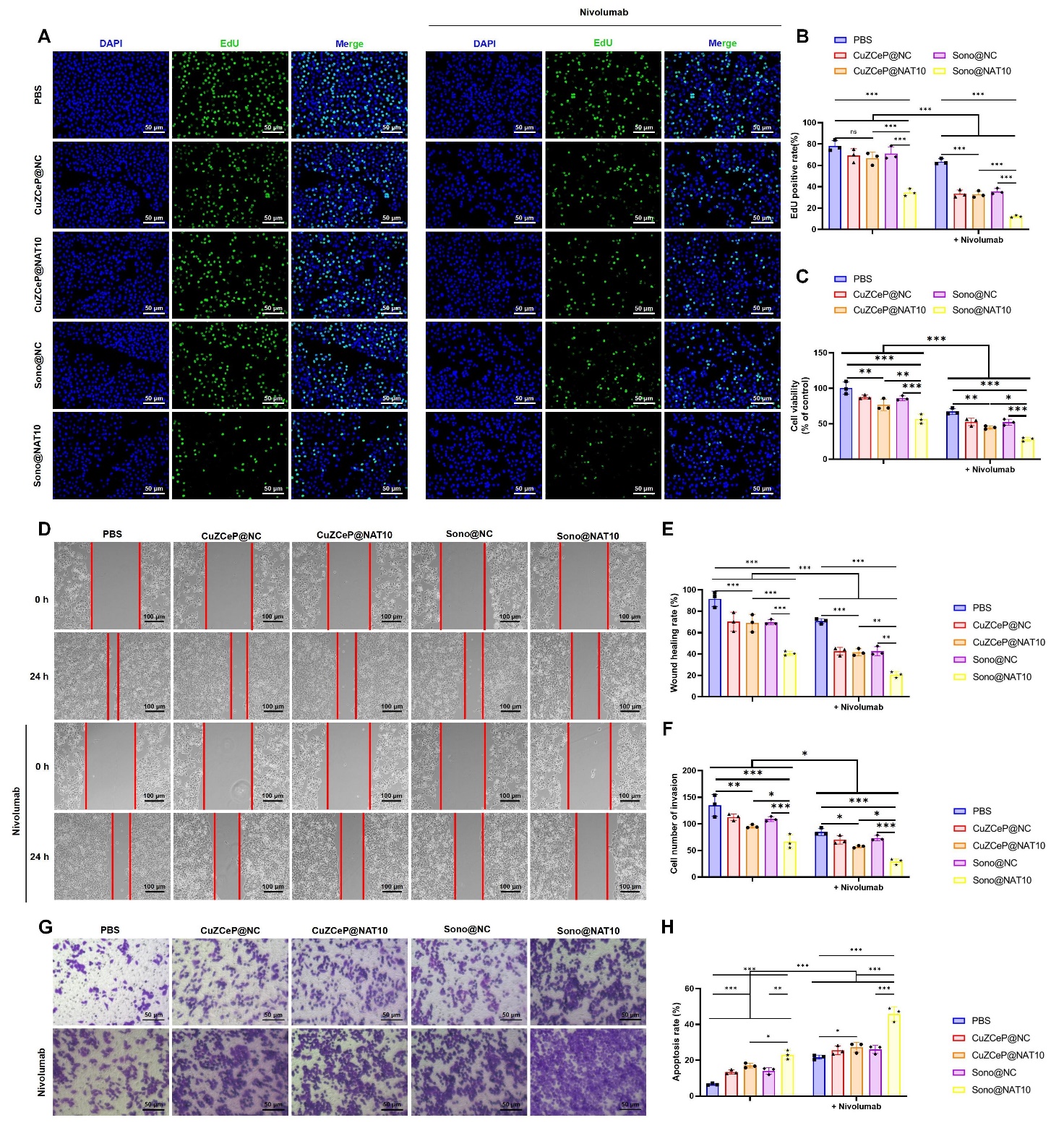


**Figure S7. Effects of Sono@NAT10 on the behavior of CMT93 cells.**

Note: (A-B) EdU assay to evaluate the proliferation capacity of CMT93 cells across groups (scale bar: 50 μm); (C) CCK-8 assay to measure cell viability in CMT93 cells; (D-E) Scratch assay to assess the migration ability of CMT93 cells (scale bar: 100 μm); (F-G) Transwell assay to analyze the invasion capability of CMT93 cells (scale bar: 50 μm); (H) Flow cytometry to detect apoptosis rates in CMT93 cells. Comparisons among the groups were performed using one-way ANOVA, and post hoc tests within groups were conducted using the Tukey method. * indicates *p <* 0.05; ** indicates *p <* 0.01. All experiments were repeated three times.


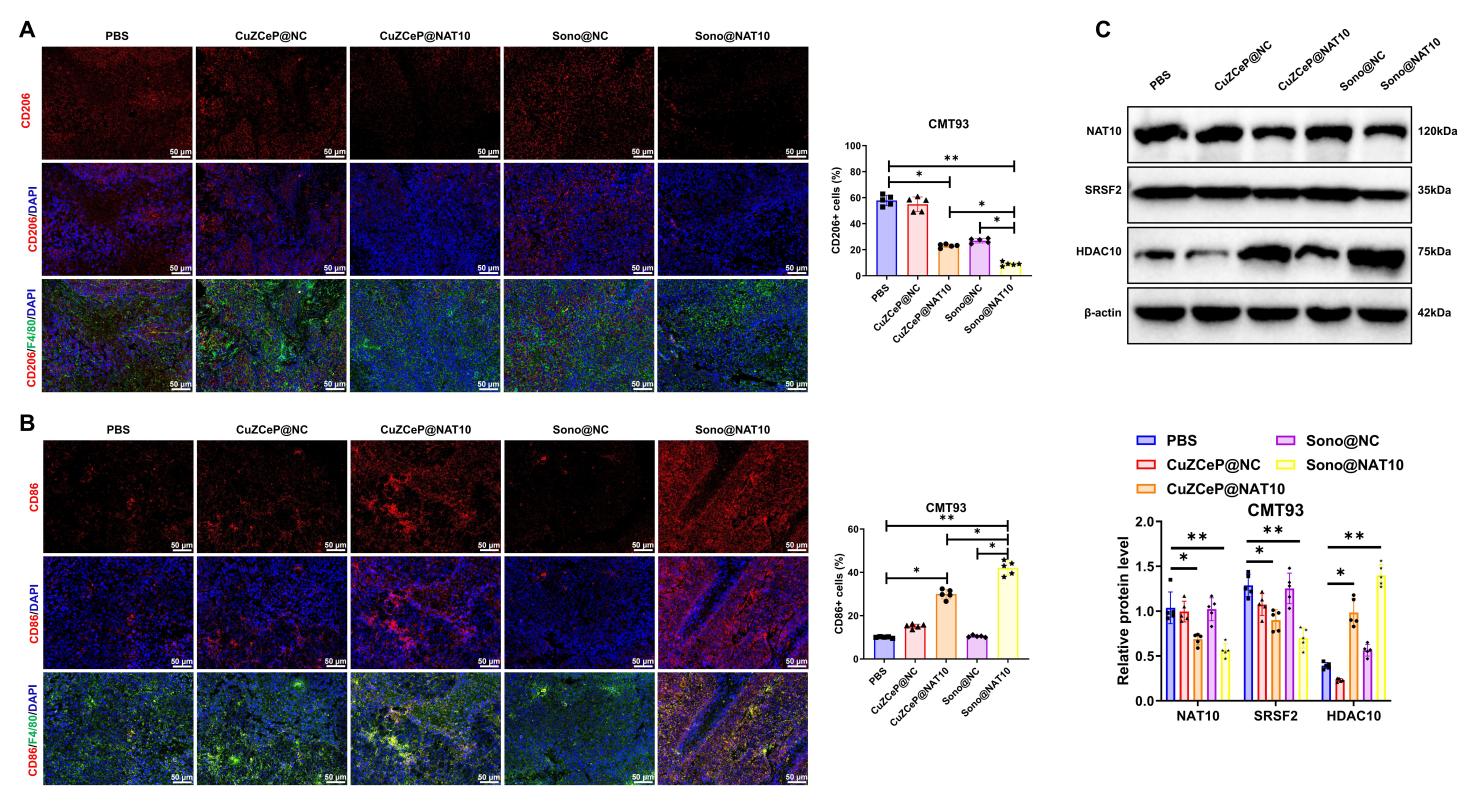


**Figure S8. Effects of nanomaterials on macrophage polarization.**

Note: (A-B) IF staining to quantify M1 macrophages (CD86) and M2 macrophages (CD206) in tumor tissues, scale bar: 50 μm; (C) Western blot analysis of NAT10, SRSF2, and HDAC10 protein levels in tumor tissues of mice across groups. Comparisons among different groups were performed using one-way ANOVA. * indicates *p <* 0.05; ** indicates *p <* 0.01. Each group included five mice.


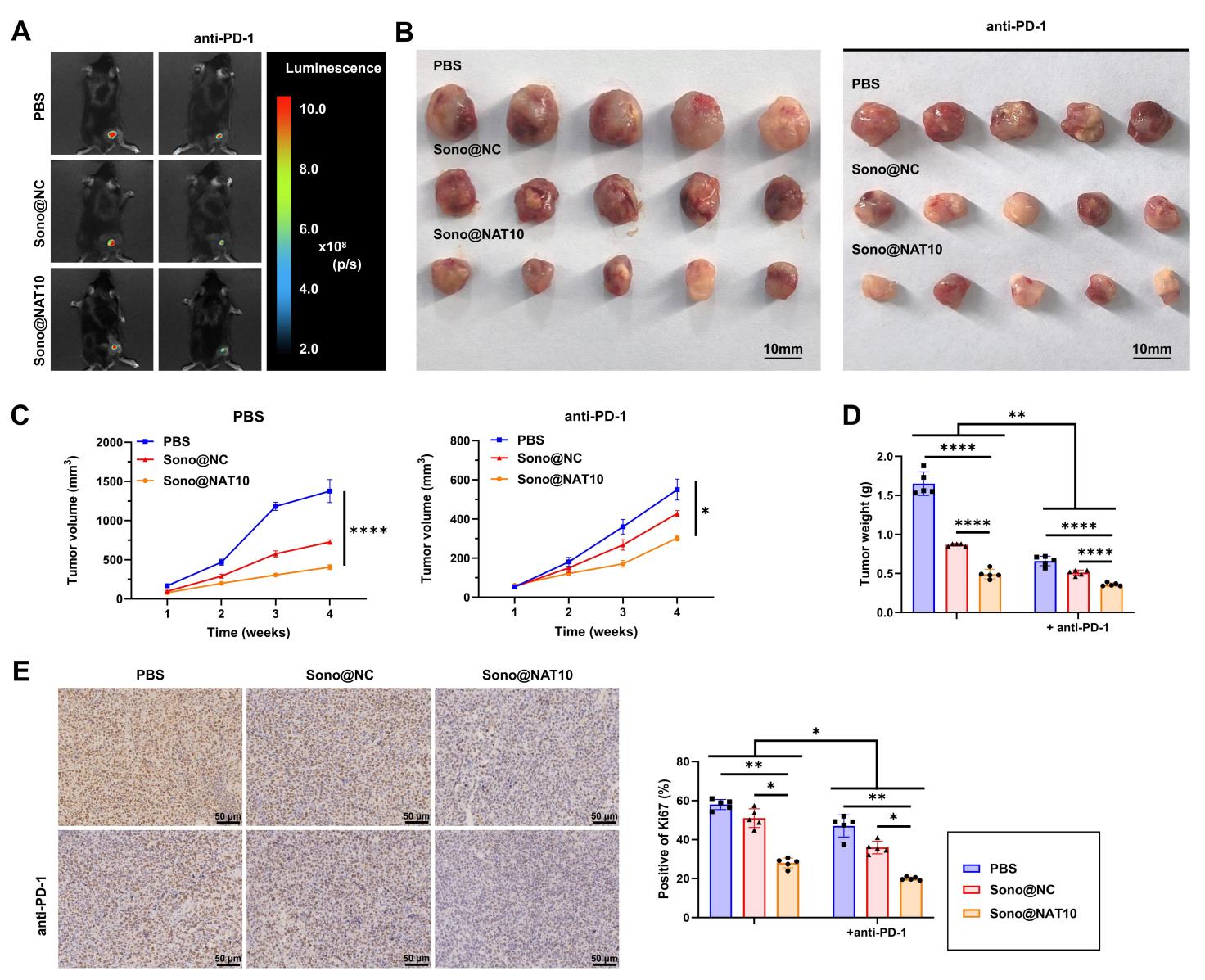


**Figure S9. Effects of nanomaterials on tumor growth in CMT93 tumor-bearing mice.**

Note: (A) Tumor growth monitored via bioluminescence intensity at different time points, with one representative example shown for each group; (B) Morphology of tumor tissues across groups; (C) Tumor growth in mice across groups; (D) Tumor tissue weights in each group; (E) Immunohistochemical staining to evaluate Ki67 protein expression levels in tumor tissues of mice across groups (scale bar: 50 μm). Comparisons of data from different groups at multiple time points and data before and after anti-PD-1 (CD279) antibody treatment among the groups were performed using two-way ANOVA, and post hoc tests within groups were conducted using the Tukey method. * indicates *p <* 0.05; ** indicates *p <* 0.01. Each group included five mice.


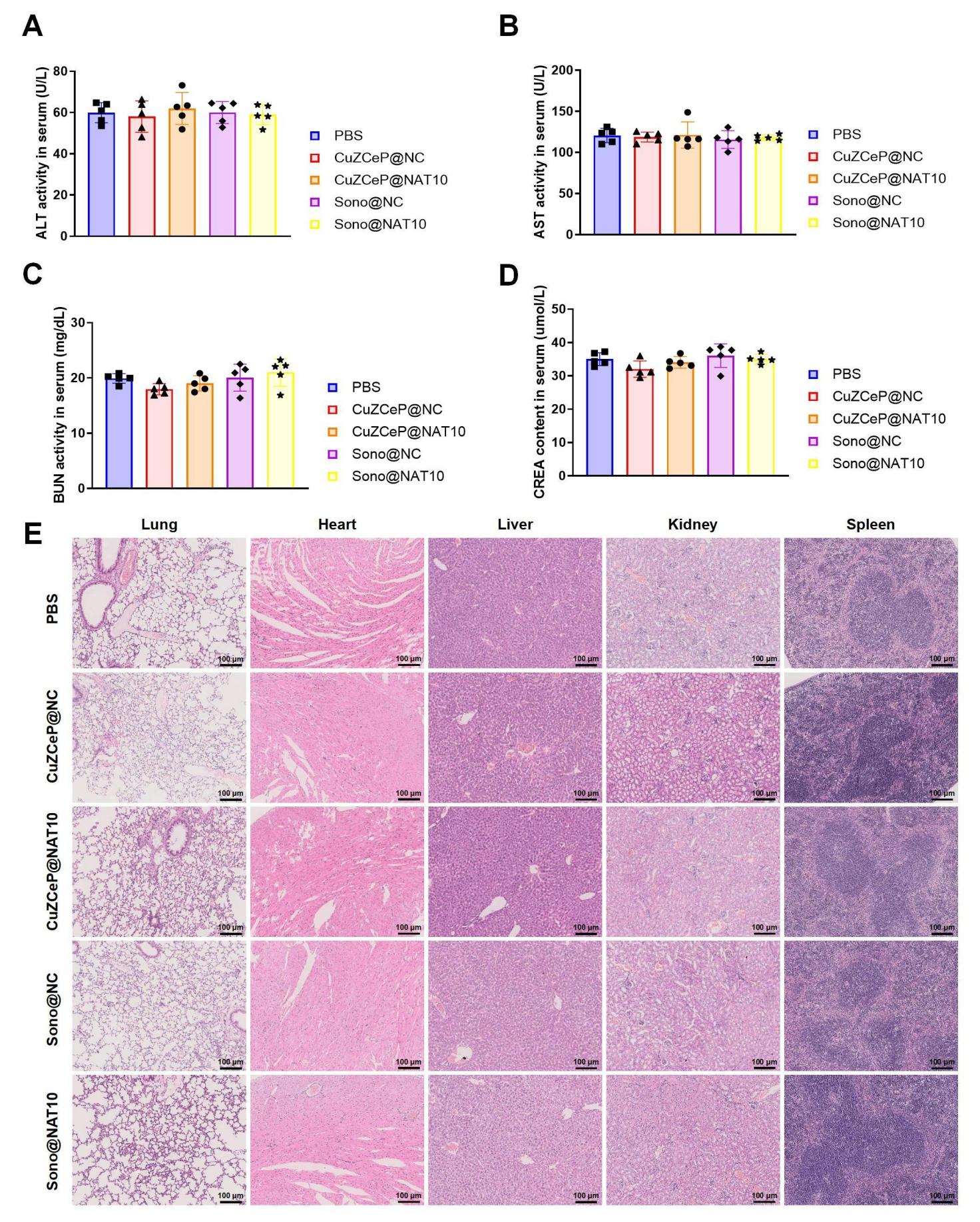


**Figure S10. Safety evaluation of Sono@NAT10 in mice.**

Note: (A-D) Effects of nanomaterials on serum ALT activity (A), AST activity (B), BUN levels (C), and CREA levels (D) across groups; (E) H&E staining of lung, heart, liver, kidney, and spleen sections in mice across groups (scale bar: 100 μm). Each group included five mice. Comparisons among different groups were performed using one-way ANOVA.

**Table S1. RT-qPCR primer sequences.**

| **Gene** | **Primer Sequence** |
| --- | --- |
| GAPDH(mouse) | F: 5'-CGGATTTGGTCGTATTGGGC-3' |
|  | R: 5'-TTGACGGTGCCATGGAATTTG-3' |
| NAT10(mouse) | F: 5'-GCGGCAGAGGTCTCTTTTTGT-3' |
|  | R: 5'-GTGACTGCTAAATCCCAGCTC-3' |
| SRSF2(mouse) | F: 5'-CGCGCTCCAGATCAACCTC-3' |
|  | R: 5'-CTTGGACTCTCGCTTCGACAC-3' |
| HDAC10(mouse) | F: 5'-ACAGCCACTCGACTGCTCT-3' |
|  | R: 5'-GATGCCTCACAAGCTGACAAA-3' |

Note: F: Forward, R: Reverse.

**Table S2. Details of antibody products.**

| **Name** | **Host** | **Target** | **Cat.** | **DiIution ratio(µg/test)** | **Manufacturer** | **Country** |
| --- | --- | --- | --- | --- | --- | --- |
| Flow cytometry antibodys | | | | | | |
| IgG | Goat | Mouse | A-11001 | 1 | Invitrogen | USA |
| F4/80 | Rat | Mouse | 11-4801-02 | 0.5 | Invitrogen | USA |
| CD206 | Rabbit | Mouse | 12-2061-82 | 0.125 | Invitrogen | USA |
| CD86 | Rabbit | Mouse | 17-0862-82 | 0.06 | Invitrogen | USA |
| Western blot antibodys | | | | | | |
| NAT10 | Rabbit | Mouse | MA5-42504 | 1:1000 | Invitrogen | USA |
| SRSF2 | Rabbit | Mouse | PA5-78164 | 1:1000 | Invitrogen | USA |
| HDAC10 | Rabbit | Mouse | MA5-32388 | 1:1000 | Invitrogen | USA |
| CD86 | Rabbit | Mouse | PA5-114995 | 1:1000 | Invitrogen | USA |
| CD206 | Rabbit | Mouse | 12-2061-82 | 1:1000 | Invitrogen | USA |
| β-actin | Rabbit | Mouse | MA1-140 | 1:5000 | Invitrogen | USA |
| IF antibodys | | | | | | |
| CD11b | Rabbit | Mouse | PA5-90724 | 1: 200 | Invitrogen | USA |
| CD206 | Rabbit | Mouse | ab64693 | 1: 200 | Abcam | UK |
| CD86 | Rat | Mouse | ab119857 | 1: 100 | Abcam | UK |
| DYKDDDDK(Flag) | Rabbit | Tag | 701629RP594 | 1: 200 | Invitrogen | USA |
| Alexa-Fluor 488 | Goat | Rabbit | ab150081 | 1: 1000 | Abcam | UK |
| Alexa-Fluor 594 | Goat | Rat | ab150160 | 1: 1000 | Abcam | UK |
| Alexa-Fluor 647 | Goat | Rabbit | ab150083 | 1: 1000 | Abcam | UK |
